# Natural, unnatural, and cause-specific mortality among current psychiatric inpatients: a systematic review and meta-analysis

**DOI:** 10.1016/j.eclinm.2025.103635

**Published:** 2025-11-14

**Authors:** Helena Angel-Scott, Ashna Basu, Allyson Tai, Pramudie Gunaratne, Patrick Bolton, Jackie Curtis, Grant Sara, Matthew Large

**Affiliations:** a Discipline of Psychiatry & Mental Health, University of New South Wales, Sydney, NSW, Australia; b The Prince of Wales Hospital, Randwick, NSW, Australia; c National Centre of Excellence in Intellectual Disability Health, UNSW Medicine & Health, Sydney, Australia; d Seriph Clinics Neuropsychiatry, St Leonards, Australia; e Mindgardens Neuroscience Network, Randwick, NSW, Australia; f Northern Clinical School Faculty of Medicine and Health University of Sydney, NSW, Australia; g System Information and Analytics Branch, NSW Ministry of Health, NSW, Australia

**Keywords:** Psychiatric inpatients, Mortality, Natural causes of death, Unnatural causes of death, Cause-specific mortality, Meta-analysis

## Abstract

**Background:**

Mortality in psychiatric hospitals is both elevated and poorly understood. We aimed to address a knowledge gap about cause-specific mortality among current psychiatric inpatients using a meta-analytic synthesis of primary research reporting on the natural, unnatural, and cause-specific mortalities.

**Methods:**

We conducted a systematic review and meta-analysis according to PRISMA and MOOSE guidelines. Searches for peer reviewed English language papers published between 1 January 1960 and 24 July 2025 and indexed in MEDLINE, PsycINFO, EMBASE and PubMed were supplemented using searches of grey literature and hand searches of reference lists. Data were pooled using random effects meta-analysis. Mortalities were assessed by cause-specific estimates of. i) the percentage of deaths, ii) mortality per 10,000 admissions and iii) mortality rates per 100,000 person-years. Temporal trends were examined using mixed effects meta-regression. A mixed effects model was used to examine subgroups within each mortality according to publication date and the national income of the study setting. This study is registered with PROSPERO (CRD42024572461).

**Findings:**

Thirty-eight studies published from diverse geographic settings over the last five decades were included in a meta-analysis of the percentage of mortality according to cause. Of these, twenty-eight studies were included in a meta-analysis of the mortality per 10,000 admissions and twenty-five studies were included in a meta-analysis of mortality rates per 100,000 patient-years. Natural causes accounted for 81.6% (95% confidence interval (CI) 75.7–86.3%, I-square = 98.5) of inpatient deaths. Vascular causes accounted for 31.0% (95% CI, 27.0–35.2%) of deaths, and infection accounted for 17.8% (CI 11.8–26.0%), The pooled estimate of natural deaths per 10,000 admissions was 78.9 (95% CI, 43.1–143.9, I-square = 99.8), falling from 246.6 (95% CI, 132.9–453.1) in pre-2000 studies to 35.2 (95% CI, 18.8–65.8) in more recent studies. The pooled estimate of the natural mortality rate per 100,000 patient-years was 2905.0 (95% CI, 2350.8–3459.2, I-square = 99.4), with rates falling from 5445.4 (95% CI, 4460.9–6429.9) in pre-2000 studies to 1374.3 (95% CI, 968.3–1780.2) in more recent studies. Suicides and accidents respectively accounted for 4.8% (95% CI, 3.0–7.7%) and 3.3% (2.3–4.7%) of deaths. Unnatural mortality rates have been stable over time. All mortality measures had high between study heterogeneity that was not well explained by the available moderators of year of publication and national income of the study setting.

**Interpretation:**

Mortality rates in psychiatric hospitals are highly heterogeneous but have been falling over time. Most inpatient deaths have a natural cause. Our findings suggest a need for primary research examining the demographic and diagnostic associations with inpatient mortality, while underscoring the opportunities for better integration of medical and mental health care.

**Funding:**

None.

**Research in context**

**Evidence before this study**

People living with major mental illness have elevated mortality rates. Historically, psychiatric research has focused on suicide. More recently, studies have highlighted the burden of mortality from natural causes among people with serious mental illness in the community, including from cardiovascular, respiratory, and metabolic diseases. Despite these insights, little is known about the rates and causes of death among current psychiatric inpatients. In an era of deinstitutionalisation and the increasing integration of psychiatric units within general hospitals, a clearer understanding of inpatient mortality could inform clinical practice, service design, and health policy. We Searched for peer reviewed English language papers indexed in MEDLINE, PsycINFO, and EMBASE using the terms ((suicid∗ and (hospital or inpatient or in-patient or admit∗) and ((mortality or outcome∗ or death∗) and (psych∗ or mental∗ or mood or affective or schizophrenia)) and (admit∗ or admis∗ or hospital∗ or inpatient∗ or in-patient∗)).mp.) and in PubMed using the terms ((Mortalit∗[Title] OR death∗[Title]) AND (hospital∗ OR admitt∗ OR inpatient∗ OR long-stay OR ward OR facility)) AND (mental∗ OR psychiatr∗ OR forensic). A search of grey literature databases was conducted in ProQuest, OpenDOAR, OAIster and the New York Academy of Medicine databases using the terms: ((Mortalit∗[Title] OR death∗[Title]) AND (hospital∗ OR admitt∗ OR inpatient∗ OR long-stay OR ward OR facility)) AND (mental∗ OR psychiatr∗ OR forensic) without language restrictions. Searches were limited to publications between 1 January 1960 and 24 July 2025. The searches were supplemented by hand searching of reference lists. Our searches resulted in the screening of 11,974 titles, 271 abstracts, and 124 full texts, of which 38 were included in the meta-analysis.

**Added value of this study**

To our knowledge, this is the first meta-analysis to quantify natural and unnatural mortality among current psychiatric inpatients. Across 38 studies, we found that mortality rates among inpatients were approximately 2.5 times higher than those observed in discharged psychiatric patients. About 80% of inpatient deaths were from natural causes. Vascular disease, infections, and respiratory illnesses emerged as the leading contributors to inpatient mortality, whereas suicide accounted for as few as 5% of deaths. The analysis also indicates that inpatient natural mortality has declined over recent decades, suggesting improvements in the general medical care of psychiatric inpatients. Nonetheless, mortality from infectious diseases is disproportionately elevated among inpatients compared with discharged patients, pointing to vulnerabilities and service gaps in infection prevention and acute physical health care within psychiatric settings.

**Implications of all the available evidence**

These findings underscore the need for a continued integration of physical and mental health care in psychiatric inpatient services. Routine screening for physical comorbidities, proactive management of cardiovascular risk factors, and robust protocols for the early recognition and treatment of medical deterioration must become core components of inpatient psychiatric care. The elevated mortality from infections highlights the need for enhanced infection control practices, including vaccination programs, systematic screening, and timely treatment interventions at the patient level. At the service level, infrastructure improvements, such as better ventilation, hygiene protocols, and closer collaboration with general medical teams, are critical. Policymakers should recognise that psychiatric inpatient facilities must be resourced and conceptualised as comprehensive health services, capable of addressing the complex interplay of mental and physical health needs in this high-risk population.

## Introduction

Reducing the mortality of people with mental illness is a critical aim of mental health care. In the last decade, there has been a growing acknowledgement of the wide mortality gap between people with mental illness and the general population^1^ and an increasing recognition that mental health services have some responsibility for physical health care.^2^ The underlying reasons for the premature mortality among the mentally ill are complex.^3^ People with mental illness have high rates of tobacco use^4^ and tend to use alcohol and other drugs^5^ that are associated with suicide, accidental death, and a range of serious illnesses. Psychiatric medications, most notably antipsychotics, are associated with metabolic syndrome,^6^^,^^7^ compounding the preexisting associations between mental illness and poor dietary habits^8^^,^^9^ and inactivity.^10^ People with mental illness also experience delays in recognition of metabolic risk factors and diagnosable illnesses,^11^ face barriers to primary and secondary prevention and have difficulties in making life-prolonging lifestyle changes.^12^^,^^13^

Historically, suicide has been the focus of mortality research among psychiatric inpatients.^14^ Less attention has been paid to medical illness and subsequent natural mortality among admitted patients. Recently, a meta-analysis of cause-specific mortality after psychiatric discharge^15^ and a large population-based study^16^ found that natural mortality, particularly vascular mortality, exceeds suicide within a few years of psychiatric discharge. Presently, it is unclear if the mortality of current inpatients differs in magnitude or its component mortalities from the mortality of psychiatrically discharged patients. To address this gap, we examined the proportion of inpatient deaths that are due to specific causes, the number of cause-specific deaths per admission, and the cause-specific mortality rates per person year. Understanding these mortalities might guide measures to improve the recognition and management of serious health conditions among psychiatric inpatients.

This study hypothesised that there have been reductions in inpatient mortality because of the mainstreaming of inpatient psychiatric care into general hospitals,^17^ reductions in length of stay,^18^ and more general progress in reducing population-wide mortality.^19^^,^^20^

## Methods

We conducted a registered systematic review and meta-analysis of observational studies reporting natural, unnatural, and total mortalities, among psychiatric inpatients, conducted according to ‘MOOSE’^21^ and PRISMA guidelines.^22^ Across each specific mortality, the extent and changes over time in the proportions of mortality, mortality per 10,000 psychiatric admissions, and mortality rates per 100,000 person-years were estimated. A study protocol was not published because the paper employed the same methods as an earlier study of cause specific mortality among discharged patients by the same research group.^15^ This study is registered with PROSPERO (CRD42024572461).

### Searches

Searches for peer reviewed English language papers indexed in MEDLINE, PsycINFO, and EMBASE using the terms ((suicid∗ and (hospital or inpatient or in-patient or admit∗) and ((mortality or outcome∗ or death∗) and (psych∗ or mental∗ or mood or affective or schizophrenia)) and (admit∗ or admis∗ or hospital∗ or inpatient∗ or in-patient∗)). mp.) and in PubMed using the terms ((Mortalit∗[Title] OR death∗[Title]) AND (hospital∗ OR admitt∗ OR inpatient∗ OR long-stay OR ward OR facility)) AND (mental∗ OR psychiatr∗ OR forensic) between 1 January 1960 and 24 July 2025, were supplemented by hand searching of reference lists. A search of grey literature databases was also conducted. ProQuest, OpenDOAR, OAIster and the New York Academy of Medicine databases were searched using the terms: ((Mortalit∗[Title] OR death∗[Title]) AND (hospital∗ OR admitt∗ OR inpatient∗ OR long-stay OR ward OR facility)) AND (mental∗ OR psychiatr∗ OR forensic), with results limited to between January 1st 1960 and 24 July 2025.

The search start year 1960 was chosen for relevance to current practice and to reflect mortality in the era of effective pharmacological treatment.^23^

The searches of title, abstract, and full text were conducted independently by HAS and ML. No methodological limitations were applied. Electronic searches were supplemented by hand-searching the reference lists of included studies.

### Inclusion and exclusion criteria

Studies were included if they reported more than one cause of death among current psychiatric inpatients in mental health facilities, including adult inpatient wards, long-stay wards, forensic wards and adolescent psychiatric wards. Papers that reported deaths shortly after transfer from inpatient psychiatric settings to medical wards, emergency departments, and intensive care units were included.

Studies were excluded if they exclusively reported mortality among patients with substance use disorders, reported on patients with a potentially confounding medical comorbidity (e.g. Huntington's Disease), reported the mortality of psychiatric outpatients or mixed inpatient and outpatients (where this data could not be separated), or reported deaths of psychiatric patients in general hospital settings. Studies that only reported on one mortality, such as suicide, were excluded to reduce the possibility of publication bias.

### Study selection and data extraction

Two authors (HAS and ML) examined full texts for inclusion, and consensus was reached by a joint examination where there was disagreement about inclusion.

HAS and AB independently extracted data from included publications into a pre-agreed spreadsheet. Where the study reported data from before 1960, only data collected post-1960 were extracted. Discrepancies were jointly reviewed and resolved by consensus. The following data were extracted: i) publication year, ii) the number of total, natural and unnatural deaths, iii) the number of deaths according to each reported specific mortality, iv) the number of admissions during the study period, and v) the total patient-years during the study period, vi) whether a country was classified as high income (HI) or low-and middle-income (LAMI) according to the World Bank classification of national income,^24^ and vii) whether English was an official language of the country in which the primary study was conducted. Where numbers of admissions were not provided, the number of admitted patients was used. Where total patient years were not reported, they were estimated by multiplying the facility bed number by the study duration.

### Coding of mortality

Specific mortalities were coded following the Cause of Death Ensemble method (CODEm).^25^ This method classifies eight causes of natural deaths (gastrointestinal, infectious, mental and behavioural, metabolic, neoplastic, respiratory, vascular, other) and three causes of unnatural deaths (accidental, homicide, suicide). Where a cause of death was not specified within CODEm classifications, ICD-11 classification^26^ was used to assign causes to CODEm categories. Where causes of death were reported using terms such as ‘other’ or ‘unspecified’, these were considered to be natural deaths. Where causes of death were reported using terms like ‘unknown’, these were included in total mortality but not in total natural or unnatural mortality. Data relating to mortalities potentially associated with mental health care that are not specified by CODEm, including deaths due to neuroleptic malignant syndrome (NMS), choking, hyponatraemia, clozapine-related deaths, electroconvulsive therapy (ECT)-related deaths, and ‘sudden’ deaths, were collected. These non-CODEm mortalities were included in the appropriate CODEm category but were also examined separately.

### Assessment of strength of reporting

A five–item scale to assess the strength of reporting was derived from quality indicators in the Newcastle–Ottawa Scale for assessing the quality of non-randomised studies in meta-analyses.^27^ Included studies were allocated with 1 point for: i) use of a recognised mortality coding system (for example, ICD 9, 10, or 11 or CODEm) to describe cause of death, ii) use of an external (non-hospital based) mortality database to ascertain mortality, iii) reporting the cause of >85% of deaths, iv) recruitment from an identified geographic catchment area, and v) examination of a study population that was not restricted by age or diagnosis. Studies were considered to have stronger reporting if they scored three or more. Data relating to study quality were independently collected by HAS and AT, with points of difference resolved by ML.

### Statistical analysis

Random effects meta-analyses were used to estimate the pooled mortality as measured by i) proportion of inpatient deaths according to cause, ii) observed mortality per admissions according to cause, and iii) mortality rates per person year according to cause. We reported deaths per admission due to its relevance to clinical practice, because this is the risk of death faced by admitted patients. We also reported rates per person year, as this is the standard measure of suicide mortality and an indication of the annual risk faced by a hospital of deaths within a psychiatric facility.

A random-effects model was chosen a priori because of the variability in patient populations and methodologies among the included studies. Between-study heterogeneity was quantified using I^2^ statistics and was explored using subgroup analysis and meta-regression. All significance tests were two-tailed. Publication bias was assessed by inspecting funnel plots and Egger's regression and was quantified using Duval and Tweedie's trim and fill method when Eggers Regression was significant at <0.05.

Random effects meta-regression (method of moments) was used to evaluate temporal changes in inpatient observed mortality per admissions and mortality rates.

Pooled subgroup estimates were compared using a mixed-effects model that did not assume a common among-study variance and a Q-value of between-group heterogeneity was used for significance testing.

Proportions of deaths were expressed as percentages, observed deaths per admission as deaths per 10,000 admissions, and mortality rates as per 100,000 patient-years.

Sensitivity analyses using a mixed-effects model were conducted according to whether the study was published before or after January 2000, and whether it was conducted in a HI or LAMI setting. Random effects meta-regression was used to examine the extent to which the total reporting strength score explained heterogeneity in the rates of total, natural and unnatural mortality per 100,000 patient-years. A further sensitivity analysis was conducted according to whether English was an official national language in the study because of the possibility of bias associated with the restriction of included studies to those written in English.

Comprehensive Meta-Analysis (CMA) Version 4 was used for all analyses.^28^ Ethical approval was not required because it was a meta-analysis of published studies. No patient consent was required.

### Role of the funding source

There was no funding source for this study.

## Results

### Sample characteristics

**Fig. 1:**
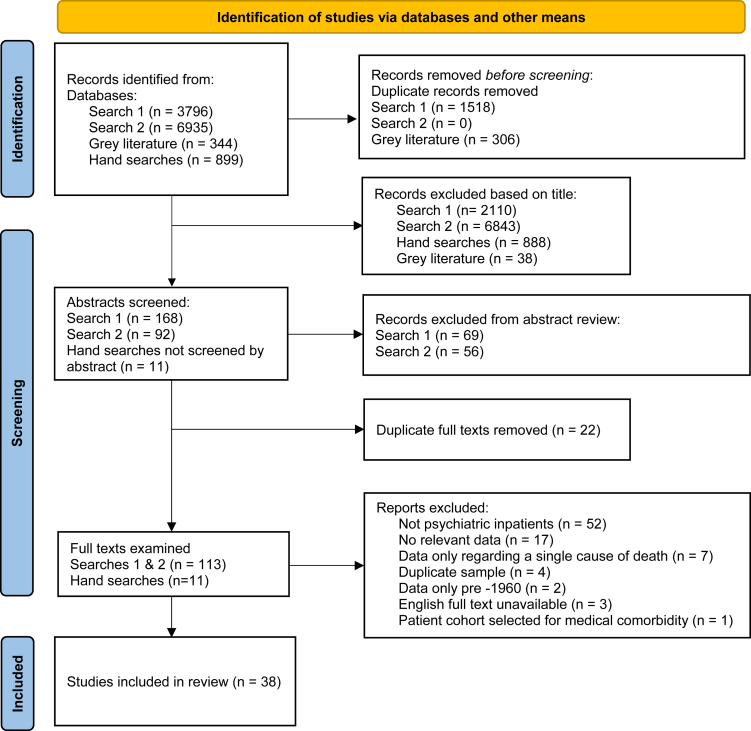
Flow chart of searches.

Thirty-eight studies met inclusion criteria^29^, ^30^, ^31^, ^32^, ^33^, ^34^, ^35^, ^36^, ^37^, ^38^, ^39^, ^40^, ^41^, ^42^, ^43^, ^44^, ^45^, ^46^, ^47^, ^48^, ^49^, ^50^^,^^51^, ^52^, ^53^, ^54^, ^55^, ^56^, ^57^, ^58^, ^59^, ^60^, ^61^, ^62^, ^63^, ^64^, ^65^, ^66^: (Fig. 1, Table 1). No study was located by the grey literature searches and all of the included studies were peer reviewed. Four studies were excluded because they reported on deaths reported in an included study.^67^, ^68^, ^69^, ^70^

**Table 1:** Table of included studies.

Study	Country	Hospital type	Patient population	Timeframe of study	Number of admissions	Patient/Bed years	Total number of deaths	Types of mortality used in meta-analysis
Aberra 2018 (Aberra, Alem et al., 2018)^29^	Ethiopia	Specialist referral hospital	All diagnoses	2006–2014	16,081	2165	40	Suicide Vascular Sudden Not Specified
Abiodun 1988 (Abiodun 1988)^30^	Nigeria	Not specified	All diagnoses	1976–1985	10,661	6971	138	Infectious Metabolic Neurological Suicide Vascular Sudden ECT
Adelugba 2018 (Adelugba, Mela et al., 2018)^31^	Canada	Forensic	All diagnoses	1997–2006	2800	2060	19	Gastrointestinal Metabolic Respiratory Suicide Vascular
Ali 2021 (Ali, Shorab et al., 2021)^32^	Egypt	Short stay unit	All diagnoses	1990–2013	17,562	2415	57	Neurological Vascular Unknown Not Specified ECT NMS Sudden
Ballinger 1976 (Ballinger and Ramsay 1976)^33^	Scotland	General adult & psychogeriatrics	All diagnoses	1971–1973	Not stated	1875	218	Infection Neoplastic Vascular Not Specified Sudden
Barbosa 2016 (Barbosa, Sequeira et al., 2016)^34^	Portugal	Acute psychiatric unit	All diagnosis	1998–2013	7772	464	21	Infectious Suicide Vascular Unknown
Black 1992 (Black and Jolley 1991)^35^	UK	General adult and psychogeriatrics	All diagnoses	1980–1985	8500	1135	100	Accident Neoplastic Respiratory Suicide Vascular Not Specified Sudden
Brook 1985 (Brook 1985)^36^	Netherlands	National sample of long stay patients	All diagnoses	1980–1981	12,139	24,278	1226	Accident Infectious Neoplastic Respiratory Suicide Vascular Unknown Not Specified
Casadebaig 1989(Casadebaig and Quemada 1989)^37^	France	National sample	All diagnoses	1982	96,751	Not Stated	6453	Accident Neoplastic Respiratory Suicide Vascular Unknown
Cengisiz 2023 (Cengisiz, Tamam et al., 2023)^38^	Turkey	Not specified	All diagnoses	2002–2022	116,202	Not Stated	120	Accident Respiratory Suicide Vascular Unknown Choking
Colton 2006 (Colton and Manderscheid 2006)^41^	Virginia, USA	Public mental health	All diagnosis	1998–2000	Not stated	20,416	261	Infectious Metabolic Neoplastic Respiratory Suicide Vascular
Costa 1981 (Costa, Mestes et al., 1981)^42^	Romania	Not specified	All diagnosis	1961–1978	Not stated	Not stated	937	Accident Gastrointestinal Infectious Metabolic Respiratory Vascular Not Specified
Craig 1983 (Craig and Lin 1983)^39^	United States	Not specified	All diagnoses	1972–1978	Not stated	Not Stated	722	Infectious Neoplastic Vascular Unknown
Craig 1984 (Craig and Lin 1984)^40^	United States	Long stay inpatients	All diagnoses	1972–1978	2743	Not Stated	880	Infectious Neoplastic Vascular Unknown
Ferriter 2016 (Ferriter, Gedeon et al., 2016)^43^	UK	Forensic patients	All diagnoses	1960–1961 1972–2000	Not stated	22,974	159	Infectious Mental & Behavioural Neoplastic Neurological Respiratory Vascular Unknown Not specified
Fulga 2021 (Fulga, Neagu et al., 2021)^44^	Romania	Involuntary patients	All diagnoses	2000–2020	Not stated	Not Stated	115	Accident Respiratory Not Specified Suicide Vascular Choking
Giel 1978 (Giel, Dijk et al., 1978)^45^	Netherlands	Long stay patients	All diagnoses	1970–1971	17,211	34,422	1506	Accident Infectious Neoplastic Respiratory Suicide Vascular Not Specified Unknown
Gunaratne 2023 (Gunaratne, Srasuebkul et al., 2023)^46^	Australia	All inpatient hospitals (statewide)	All diagnosis	2002–2012	421,580	11,550	471	Neoplastic Neurological Mental & Behavioural Respiratory Suicide Vascular Not Specified Unknown
Hewer 1995 (Hewer, Rössler et al., 1995)^47^	Germany	Hospitals with a catchment area	All diagnoses	1984–1986	14,195	1917	196	Metabolic Neoplastic Neurological Respiratory Suicide Vascular Not Specified Unknown
Hwang 1998 (Hwang, Tsai et al., 1998)^48^	Taiwan	General adult and veteran	All diagnoses	1978–1995	1208	88	18	Accident Gastrointestinal Infectious Metabolic Neoplastic Neurological Respiratory Vascular NMS
Ifteni 2014 (Ifteni, Correll et al., 2014)^49^	Romania	Not specified	Schizophrenia	1989–2013	7189	3000	57	Accident Neurological Respiratory Vascular Unknown Choking
Kamara 1998 (Kamara, Peterson et al., 1998)^50^	USA	State hospital	All diagnoses	1989–1994	9437	6102	179	Gastrointestinal Infectious Mental & behavioural Metabolic Neoplastic Neurological Respiratory Vascular Not specified
Kaggwa 2021 (Kaggwa, Najjuka et al., 2021)^51^	Uganda	Regional Hospital	All diagnoses	1995–2020	Not stated	Not Stated	43	Accident Infectious Metabolic Respiratory Vascular Unknown
Khamker 2010 (Khamker, Moola et al., 2010)^52^	South Africa	Not specified	All diagnoses	2001–2005	11,131	4870	164	Accident Gastrointestinal Infectious Metabolic Neoplastic Neurological Respiratory Suicide Vascular Unknown Choking Sudden
Khlil 2023 (Khlil, Attouche et al., 2023)^53^	Morocco	Not specified	All diagnoses	2011–2021	9038	1144	18	Accident Gastrointestinal Neurological Suicide Vascular Unknown Choking NMS Sudden
Lim 1991 (Lim, Sim et al., 1991)^54^	Singapore	Not specified	All diagnoses	1985–1986	3984	4600	120	Accident Homicide Infectious Neoplastic Suicide Vascular Not Specified Choking
Malomo 2003 (Malomo, Aina et al., 2004)^55^	Nigeria	Not specified	All diagnoses	1991–2000	11,365	5350	96	Gastrointestinal Infectious Metabolic Neoplastic Neurological Respiratory Suicide Vascular Not Specified NMS Sudden
Ndosi 1997 (Ndosi and Kisesa 1997)^56^	Tanzania	Not specified	All diagnoses	1990–1994	7868	300	146	Accident Gastrointestinal Infectious Metabolic Neoplastic Neurological Vascular Unknown Choking
Ojansuu 2018(Ojansuu, Putkonen et al., 2018)^57^	Finland	Forensic	All diagnoses	1980–2009	1253	7393	96	Accident Homicide Suicide All Natural
Osman 2018 (Osman, Abubakker et al., 2018)^58^	Sudan	Not specified	All diagnosis	2001–2009	20,745	Not Stated	72	Infectious Metabolic Neurological Suicide Vascular Unknown ECT NMS Sudden
Osman 2020 (Osman, Abdalhai et al., 2020)^59^	Sudan	Not specified	All diagnoses	2011–2015	7340	Not Stated	36	Infectious Gastrointestinal Mental & Behavioural Metabolic Neurological Respiratory Vascular ECT NMS Sudden
Pérez-Cárceles 2001 (Pérez-Cárceles, Íñigo et al., 2001)^60^	Spain	Forensic	All diagnoses	1984–1997	92,754	3024	64	Gastrointestinal Homicide Metabolic Neoplastic Respiratory Suicide Vascular
Saito 2014 (Saito, Kobayashi et al., 2014)^61^	Japan	Not specified	Eating disorder	2005–2012	111	328	3	Gastrointestinal Infectious
Saugstad 1979 (Saugstad and Odegard 1979)^62^	Norway	Not specified	All diagnoses	1962–1974	123,578	Not stated	6151	Gastrointestinal Infectious Metabolic Neoplastic Respiratory Vascular Accident
Shinde 2014 (Shinde, Nagarajaiah et al., 2014)^63^	India	Not specified	All diagnoses	1983–2008	103,252	Not Stated	333	Accident Gastrointestinal Infectious Metabolic Neurological Respiratory Suicide Vascular Unknown Not Specified
Shinozaki 1976 (Shinozaki 1976)^64^	Japan	Not specified	All diagnoses	1968–1970	30,597	Not Stated	1267	Accident Gastrointestinal Infectious Mental & behavioural Metabolic Neoplastic Neurological Respiratory Suicide Vascular Unknown
Swain 2019 (Swain and Behura 2019)^65^	India	Tertiary Care Hospital	All diagnoses	1998–2018	139,304	Not Stated	151	Gastrointestinal Metabolic Neurological Respiratory Vascular Unknown
Wu 2022 (Wu, Xia et al., 2022)^66^	China	Non-forensic province hospitals	All diagnoses	2019–2020	Not stated	Not Stated	719	Neoplastic Respiratory Suicide Not Specified Unknown Sudden

The 38 papers reported 23,372 deaths according to cause (median number of deaths per study = 142, interquartile range 57–595). 28 of the 38 papers reported the number of admissions and were meta-analysed to estimate the mortality per 10,000 admissions. 25 of the 38 papers reported the total patient-years meta-analysed to estimate rates per 100,000 patient-years. The studies reported a mean number 5.4 (SD = 2.0) of the 11 CODEm mortalities.

Publication dates ranged from 1976 to 2023, with a median date of 2005. Study duration ranged from 1 to 30 years (mean = 11.7, SD = 20.5, Median = 9.5). The mean interval between the midpoint of the study period and the year of publication was 10.4 years (SD = 5.6).

The studies had diverse national origins from Europe, North America, Africa, Asia, the Middle East, and Oceania. Four studies were from the United States of America, three from the United Kingdom and three from Romania. Two studies were respectively from India, Japan, the Netherlands, Nigeria, and Sudan. One study was reported from each of Australia, Canada, China, Egypt, Ethiopia, Finland, France, Germany, Morocco, Norway, Portugal, Singapore, South Africa, Spain, Taiwan, Tanzania, Turkey and Uganda. Twenty-three papers were from HI countries, and 15 were from LAMI countries. Nine studies were from predominantly English-speaking countries, and a further six studies were from countries where English is an official language used in government and education.

Seventeen of 38 studies reported on the gender mix of the patient population, and four studies reported on the age distribution. Eight of 38 studies reported information about the diagnostic mix of the patient groups. Six studies examined specific patient groups; three assessed long-stay inpatients, one examined people over sixty-five years, one examined eating disorders, and one examined people with schizophrenia. Four studies reported mortality in forensic wards and two studies combined general adult and psychogeriatric wards. One study excluded forensic wards, and one specified general adult wards.

### Strength of reporting

Results of the assessment of study strength can be found in Supplementary Material 1 (Supplementary Table S1. Strength of reporting scores). The median strength of reporting score was 3 out of 5, with 21 of 38 included studies scoring at or above this value. Thirteen studies used a recognised diagnostic system to classify the cause of death, ten used an external mortality database to confirm inpatient deaths, 24 reported the cause of ≥85% of deaths, 22 were conducted in an identifiable catchment area, and 35 examined patients who were not selected by age or diagnosis.

None of the five reporting strength items were explanatory of between study heterogeneity in total mortality or total natural mortality rates, and there was no interaction between reporting strength items and publication date. The total reporting strength was unrelated to total natural mortality rates per 100,000 person-years (slope = 0.006, standard error of slope = 0.002, z-value = 0.26, P = 0.79), or total mortality (slope = −0.0003, z-value = −1.00, P-value = 0.91), and there were no significant interactions between reporting strength and date of publication in a multivariate analysis. Higher quality studies reported a higher total unnatural mortality rate per 100,000 person years (slope = 0.001, z-value = 3.66, P-value = 0.0003). Studies that used an external mortality data base reported a significantly higher suicide mortality of 299 (95% CI, 168–430) suicides per 100,000 person years when compared to studies that ascertained suicide using local data (54 suicides per 100,000, 95% CI, 22–86: Q-value = 12.75, df (q) = 1, P-value = <0.0001).

### Mortalities

Total mortality occurred at a frequency of 111.6 per 10,000 (95% CI = 68.4–181.6) admissions (equivalent to one death per 89.6 admissions) at a rate of 3919 (95% CI = 3133.2–4704.8) deaths per 100,000 patient-years. The leading causes of death, according to the proportion of all mortality, were vascular (31.0%), infectious (17.8%), and respiratory (14.0%). Unspecified causes comprised 10.6% of deaths, and the cause of 13.8% was unknown to the primary researchers. Suicide resulted in 4.8% of deaths (Table 2: Proportions of Specific Cause Mortality Among Psychiatric Inpatients). The leading causes of deaths per 10,000 admissions were infectious (34.2), vascular (25.1), neoplastic (13.2), and respiratory (11.7). Sudden death ended 7.8 per 10,000 admissions (Table 3. Specific Cause Specific Mortality per 10,000 Psychiatric Admissions). The leading cause of deaths per 100,000 person years were vascular (953.7), infectious (617.6), respiratory (396.8), neoplastic (191.2), mental and behavioural (166.8) neurological (149.7), suicide (139.9), gastrointestinal (112.8), and accidents (92.1) (Table 4 Deaths Per 100,000 Person Years among psychiatric inpatients).

**Table 2:** Mortalities in psychiatric hospitals by proportions.

	Pooled estimate				Heterogeneity	Egger's regression			Meta-regression on date			
	n. Studies	Estimate (%)	Lower limit (%)	Upper limit (%)	I-square	Intercept	SE	P-value	Coefficient	SE	Z-value	P-value
Gastrointestinal	14	5.3%	3.3%	8.4%	82.0	1.44	1.24	0.270	0.03	0.015	2.02	0.04
Infectious	22	17.8%	11.8%	26.0%	97.8	−1.81	2.57	0.790	<0.001	0.017	0.05	0.96
Ment. & Beh.	5	3.6%	2.0%	6.4%	81.9	−2.04	1.60	0.270	0.008	0.020	0.42	0.67
Metabolic	17	3.6%	2.2%	6.1%	84.3	−1.36	1.40	0.350	0.016	0.020	0.79	0.43
Neoplastic	22	6.6%	5.6%	7.9%	86.7	−1.25	0.85	0.160	<0.001	0.006	−0.01	0.99
Neurological	17	6.8%	5.2%	8.9%	60.5	−1.91	0.67	0.010	0.034	0.011	2.930	0.003
Respiratory	24	14.0%	9.7%	19.8%	98.2	−0.44	2.37	0.850	−0.004	0.013	−0.260	0.790
Vascular	35	31.0%	27.0%	35.2%	96.7	1.48	1.31	0.270	0.005	0.006	0.880	0.380
Not specified	15	10.6%	6.8%	16.1%	97.2	−4.61	2.11	0.048	0.026	0.015	1.710	0.090
**Total natural**	**37**	**81.6%**	**75.7%**	**86.3%**	**98.5**	**6.56**	**1.35**	**<0.0001**	**−0.011**	**0.110**	**−0.930**	**0.350**
Accident	19	3.3%	2.3%	4.7%	92.3	−0.02	0.01	0.11	0.015	0.011	1.41	0.16
Homicide	3	1.7%	0.6%	4.4%	0.0	−4.30	0.57	0.08				
Suicide	25	4.8%	3.0%	7.7%	96.3	1.03	1.61	0.53	0.019	0.013	1.460	0.140
**Total unnatural**	**37**	**7.1%**	**5.4%**	**9.2%**	**94.3**	**−0.39**	**0.94**	**0.68**	**0.016**	**0.007**	**2.240**	**0.025**
Unknown	22	13.8%	9.1%	20.3%	98.6	−1.54	2.73	0.58	<−0.001	0.015	−0.040	0.970
Sudden	11	14.1%	9.0%	21.4%	87.7	0.01	1.78	0.99	0.012	0.018	0.350	0.510
Choking	7	4.3%	1.5%	11.3%	85.4	−0.05	0.01	0.009	0.045	0.042	1.06	0.29
ECT	4	2.0%	0.8%	4.8%	0.0	−3.17	2.22	0.29	0.043	0.035	1.22	0.22
NMS	6	6.4%	2.7%	14.2%	57.9	−2.71	0.96	0.047	0.058	0.050	1.170	0.240

**Table 3:** Mortalities in psychiatric hospitals per 10,000 admissions.

	Pooled estimate				Heterogeneity	Egger's regression			Meta-regression on date			
	n. Studies	Estimate	Lower limit	Upper limit	I-square	Intercept	SE	P-value	Coefficient	SE	Z-value	P-value
Gastrointestinal	12	4.5	2.2	9.4	87.8	−1.51	1.68	0.390	−0.036	0.0300	−1.17	0.24
Infectious	15	34.2	16.1	72.5	99.1	−9.50	4.14	0.039	−0.067	0.0270	−2.51	0.012
Ment. & Beh.	5	2.3	0.5	11.1	98.0	−3.02	6.46	0.670	−0.061	0.0040	−13.9	<0.0001
Metabolic	13	3.9	2.0	7.8	89.6	−3.41	2.1	0.130	−0.044	0.0240	−1.8	0.07
Neoplastic	14	13.2	7.0	24.6	98.8	−5.08	3.16	0.130	−0.085	0.0240	−3.55	0.0004
Neurological	16	3.4	2.0	6.0	89.5	1.14	1.57	0.480	−0.032	0.0180	−1.83	0.07
Respiratory	16	11.7	6.0	23.0	99.0	−8.44	3.21	0.020	−0.081	0.0130	−6.35	<0.0001
Vascular	26	25.1	14.1	44.5	99.7	−11.80	4.37	0.010	−0.077	0.0150	−5.13	<0.0001
Not specified	10	7.3	1.4	39.1	99.5	−12.51	5.68	0.058	−0.058	0.0340	−1.7	0.09
**Total natural**	**27**	**78.9**	**43.1**	**143.9**	**99.8**	**−14.60**	**6.49**	**0.030**	**−0.079**	**0.0140**	**−5.7**	**<0.0001**
Accident	14	6.5	3.4	12.7	97.5	−5.31	1.74	0.010	−0.044	0.0230	−1.9	0.058
Homicide	2	4.5	1.1	17.9	0.0							
Suicide	20	5.4	2.9	9.8	97.5	−2.24	2.15	0.310	−0.044	0.0190	−2.32	0.02
**Total unnatural**	**27**	**7.7**	**4.5**	**13.2**	**98.5**	**−4.09**	**1.93**	**0.040**	**−0.065**	**0.0160**	**−4.17**	**<0.0001**
Unknown	16	9.9	4.1	23.9	99.6	−13.60	4.32	0.007	−0.104	0.0200	−5.12	<0.0001
Sudden	9	7.8	4.3	14.4	87.7	−5.67	1.2	0.002	−0.038	0.0250	−1.51	0.13
Choking	6	2.1	0.6	7.4	83.0	−6.40	2.99	0.100	−0.068	0.0430	−1.58	0.11
ECT	4	2.1	0.8	5.5	50.4	−2.44	0.58	0.052	0.024	0.0440	0.54	0.59
NMS	6	3.0	1.6	5.8	29.6	−1.58	0.93	0.170	−0.01	0.0440	−0.23	0.81
**Total deaths**	**26**	**111.6**	**68.4**	**181.6**	**99.9**	**−20.10**	**6.61**	**0.005**	**−0.077**	**0.0120**	**−6.41**	**<0.0001**

**Table 4:** Mortalities in psychiatric hospitals per 100,000 person years.

	pooled estimate				Heterogeneity	Egger's regression			Meta-regression on date			
	n. Studies	Estimate	Lower limit	Upper limit	I-square	Intercept	SE	P-value	Coefficient	SE	Z-value	P-value
Gastrointestinal	11	112.8	54.1	171.5	86	2.43	0.45	0.0004	<−0.0001	<0.0001	−0.27	0.78
Infectious	15	617.6	483.1	752.1	98	7.22	1.37	0.0002	−0.0002	0.0001	−3.51	0.0005
Ment. & Beh.	4	166.8	37.7	296.0	96	6.28	2.07	0.09	<−0.0001	<0.0001	−0.19	0.85
Metabolic	11	40.4	18.7	62.1	85	2.55	0.29	0.0001	<−0.0001	<0.0001	−1.01	0.31
Suicide	17	139.9	100.5	179.3	94	3.69	0.73	0.0002	<0.0001	<0.0001	1.93	0.054
Neoplastic	16	191.2	130.5	251.8	95	4.04	0.7	<0.0001	<−0.0001	<0.0001	−1.72	0.09
Neurological	13	149.7	84.9	214.6	89	2.98	0.49	<0.0001	<−0.0001	<0.0001	−2.08	0.04
Respiratory	15	396.8	310.1	483.5	98	6.32	1.33	0.0004	−0.0002	<0.0001	−5.04	<0.0001
Vascular	23	953.7	730.7	1176.8	99	7.11	1.21	<0.0001	−0.0003	<0.0001	−3.24	0.001
Not specified	12	547.5	331.7	763.3	98	6.52	1.82	0.005	<−0.0001	<0.0001	−0.66	0.51
**Total natural**	**25**	**2905.0**	**2350.8**	**3459.2**	**99**	**11.55**	**1.7**	**<0.0001**	**−0.001**	**0.0002**	**−5.32**	**<0.0001**
Accident	12	92.1	43.7	140.5	87	2.67	0.64	0.002	<−0.0001	<0.0001	−0.88	0.38
Homicide	3	2.4	0.0	5.4	0	0.96	0.01	0.007				
Suicide	17	139.9	100.4	179.3	94	3.69	0.734	0.0002	<0.0001	<0.0001	1.93	0.053
**Total unnatural**	**24**	**184.7**	**139.2**	**230.2**	**94**	**3.54**	**0.74**	**0.0001**	**<0.0001**	**<0.0001**	**0.91**	**0.36**
Unknown	13	719.0	457.4	980.5	99	8.35	2.71	0.01	−0.0003	<0.0001	−7.19	<0.0001
Sudden	8	394.7	204.3	585.1	91	4.03	0.65	<0.0001	<−0.0001	<0.0001	−1.81	0.07
Choking	5	72.9	28.0	117.8	0	1.38	0.43	0.049	<−0.0001	<0.0001	−0.17	0.86
ECT	2	17.2	0.0	43.8	0							
NMS	4	32.9	0.0	84.1	18	1.32	0.29	0.046	<0.0001	<0.0001	0.57	0.57
**Total deaths**	**24**	**3919.0**	**3133.2**	**4704.8**	**100**	**13.29**	**2.28**	**<0.0001**	**−0.0013**	**0.0003**	**−4.95**	**<0.0001**

Total mortality has fallen according to both deaths per admission and deaths per patient-year. Among studies published before the year 2000, there were 359.7 (95% CI, 246.2–552.7) deaths per 10,000 admissions associated with 7955.1 (95% CI, 6391.8–9518.2) deaths per 100,000 person years, while among studies published in or after the year 2000, there were 46.4 (95% 24.1–89.1) deaths per 10,000 admissions associated with 1806.8 (95% CI, 1267.1–2346.5) deaths per 100,000 person years (Supplementary Tables S3 and S4).

### Natural mortalities

81.6% of deaths had natural causes, with natural deaths occurring at an observed 78.9 deaths per 10,000 admissions (one death per 127 admissions), and a rate of 2905 deaths per 100,000 patient-years. Total natural deaths declined over time as measured by both deaths per 10,000 admissions (Table 3) and deaths per 100,000 patient years (Table 4). Natural deaths per admission fell from 246.6 (95% CI, 132.9–453.2) per 10,000 admissions in studies published before 2000 to 35.2 (95% CI, 18.8–65.9) in later studies (Supplementary Table S3). The natural mortality rate fell from 5445 (95% CI, 4460.9–6429.9) in pre-2000 studies to 1374 (95% CI, 968.3–1780.2) in later studies (Supplementary Table S4). There was evidence of publication bias against studies with a higher mortality, and Duval and Tweedie's trim and fill identified three studies to the right of the mean and provided a revised estimate of 4333 95% (95% CI, 2620–6064) deaths per 100,000 person years. The removal of one study with an outlying rate of total death of 37,700 deaths per 100,000 patient years (almost all of which was infective) reported from an overcrowded ward during the AIDS epidemic in Tanzania in the 1990's^56^ reduced the estimated rate of total natural death from 2905 to 2704 (95% CI, 2155–3253) deaths per 100,000 patient years.

**Fig. 2:**
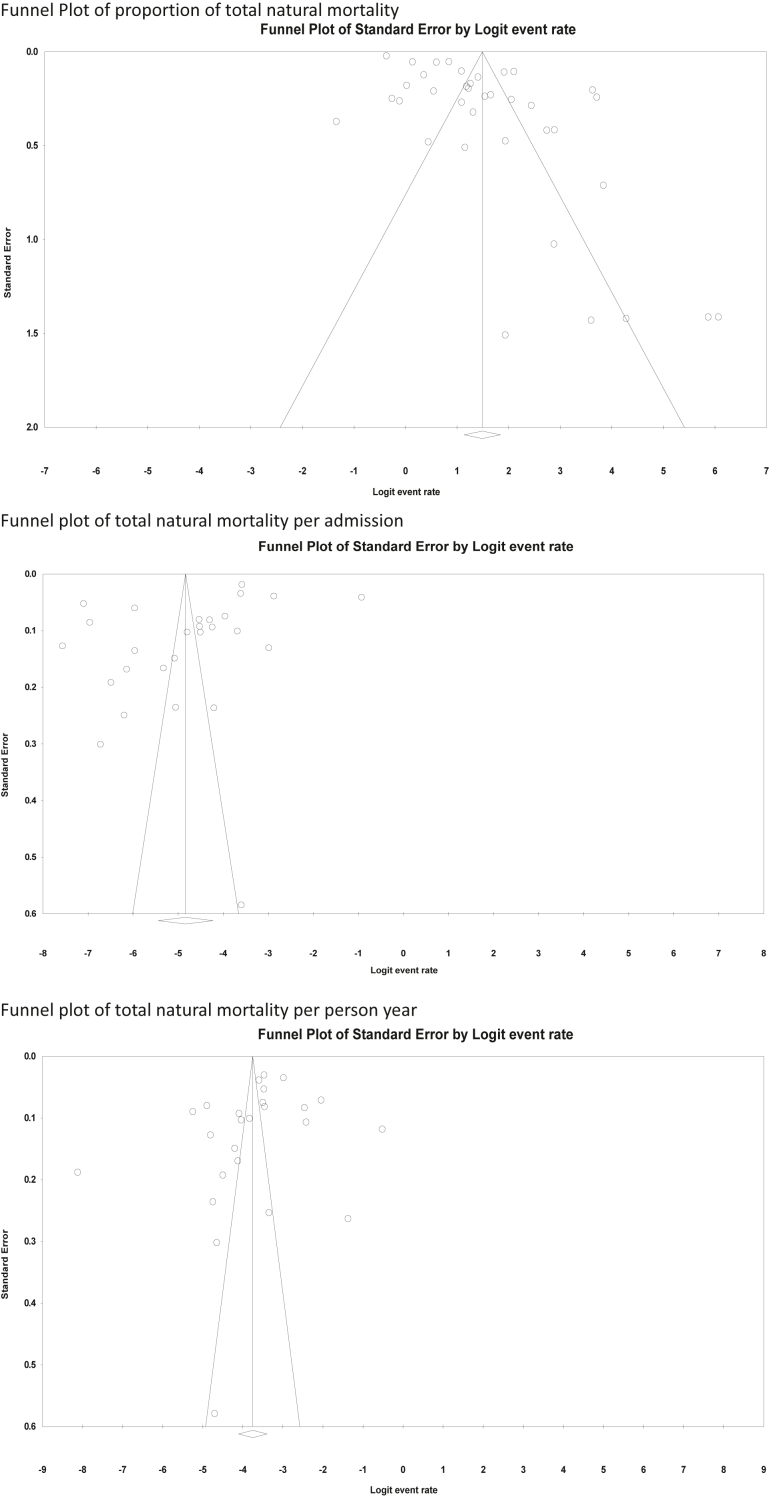
Funnel plots of total natural mortality.

Funnel plots of the proportion of natural deaths, deaths per admission, and the rate of death per patient year can be seen in Fig. 2. The primary analyses, tests of publication bias, and meta-regression with the moderator of year of publication can be found according to proportion of deaths (Table 2), deaths per admission (Table 3), and deaths per patient year (Table 4).

There was evidence of publication bias towards studies with a higher proportion of natural deaths, as evidenced by the funnel plot and significant Eggers Regression (Table 2). Duval and Tweedie's trim and fill identified seven hypothetically missing studies to the left of the mean and resulted in a reduced estimate of the proportion of natural deaths of 73.7% (95% CI 66.5–79.7%).

There was evidence of publication bias in natural mortality per admission (Table 3). However, Duval and Tweedie's trim method provided an unchanged estimate of the number of natural deaths per admission.

There was evidence of publication bias towards a lower reported total natural mortality per person-year (Table 4). Duval and Tweedie's trim method identified three hypothetically missing studies to the right of the mean and calculated an increased estimate from 2905 to 4333 (95% CI 2620–6046) deaths per 100,000 person years.

**Fig. 3:**
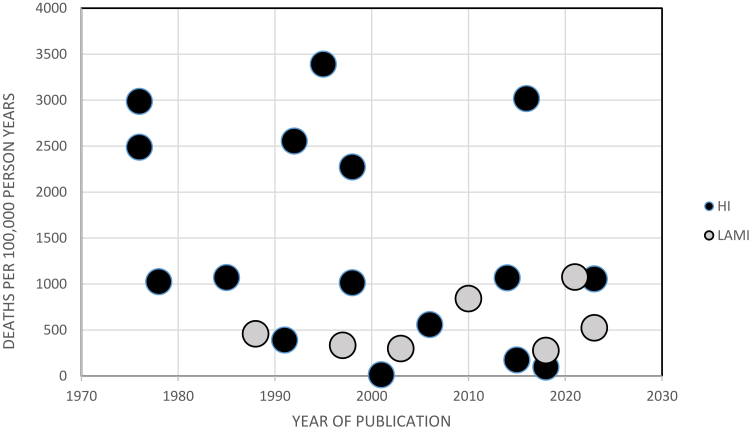
Vascular mortality per 100,000 person years.

Vascular mortality caused 31.0% of all deaths and occurred at an observed frequency of 25.1 deaths per 10,000 admissions, at a rate of 954 deaths per 100,000 person-years. Vascular deaths declined over time, as measured by both deaths per 10,000 admissions and the rate per person per year (See Fig. 3, Vascular Mortality per 100,000 person years, Supplementary Tables S3 and S4). There were no outlying studies, and Duval and Tweedie's trim and fill did not suggest a revised estimate.

**Fig. 4:**
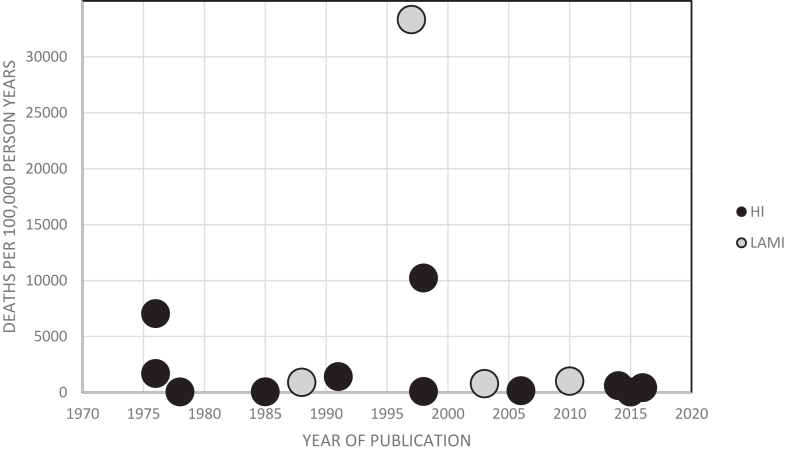
Infectious mortality per 100,000 person years.

Infectious deaths were responsible for 17.8% of all mortality and occurred at an observed frequency of 34.2 deaths per 10,000 admissions and a rate of 618 deaths per 100,000 patient-years. Both infectious deaths per 10,000 admissions and infectious deaths per 100,000 person-years demonstrated a decline over time (see Fig. 4: Infectious Mortality per 100,000 person-years). The removal of one study with an outlying rate of infectious deaths of 33,000 deaths per 100,000 patient years reported from Tanzania in the 1990s^56^ reduced the estimated rate of infectious deaths from 618 to 586 (95% CI, 459–712) deaths per 100,000 patient years. Duval and Tweedie's trim and fill identified two hypothetically missing studies and provided a minimally reduced estimate from 618 to 615 (95% CI 473–757) deaths per 100,000 person years.

Respiratory deaths comprised 14.0% of total mortality, with an observed frequency of 11.7 deaths per 10,000 admissions and occurred at a rate of 397 deaths per 100,000 patient-years. There was evidence of publication bias in favour of studies with a high rate of respiratory death, and Tweedie and Duval trim and fill identified three hypothetically missing studies and provided a revised estimate of 282 (95% CI 194–371) deaths per 100,000 person years. Respiratory deaths declined over time as measured by both deaths per 10,000 admissions and deaths per 100,000 (Supplementary Tables S3 and S4).

Neurological deaths represented 6.8% of all deaths and occurred at an observed frequency of 3.4 deaths per 10,000 admissions, at a rate of 149.7 deaths per 100,000 patient-years. A decline was observed in neurological deaths per 10,000 admissions, but deaths on a per-person-year basis were unchanged.

Neoplastic deaths comprised 6.6% of all deaths, with 13.2 deaths per 10,000 admissions, and at a rate of 191 deaths per 100,000 person-years. There was evidence of publication bias in favour of studies with a high rate of neoplastic death, and Tweedie and Duval trim and fill identified four hypothetically missing studies and provided a revised estimate of 168 (95% CI 108–2227) deaths per 100,000 person years. Neoplastic deaths showed a declining trend over time as measured by deaths per 10,000 admissions and the rate of deaths per 100,000.

Gastrointestinal deaths accounted for 5.3% of all deaths, occurring at a frequency of 4.5 deaths per 10,000 admissions and a rate of 113 deaths per 100,000 person-years. There was evidence of publication bias in favour of studies with a high rate of gastrointestinal death, and Tweedie and Duval trim and fill identified four hypothetically missing studies and provided a revised estimate of 97 (95% CI 40–154) deaths per 100,000 person years. There was no evidence of a significant change over time in the number of gastrointestinal deaths per 10,000 admissions or in the rate of gastrointestinal deaths per 100,000.

Metabolic causes accounted for 3.6% of all deaths and occurred at an observed frequency of 3.9 deaths per 10,000 admissions, at a rate of 40.4 deaths per 100,000 person-years. These rates remained stable over time, with no evidence of a significant change in the number of metabolic deaths per 10,000 admissions or in the rate of deaths per 100,000 person years.

Mental and behavioural disorders accounted for 3.6% of all deaths, occurring at a frequency of 2.3 deaths per 10,000 admissions and a rate of 167 deaths per 100,000 person-years. Five studies referenced mental and behavioural causes of death in which the most common cause was delirium tremens.

### Unnatural mortality

Unnatural deaths accounted for 7.1% of mortality and occurred at an observed frequency of 7.7 deaths per 10,000 admissions, at a rate of 185 deaths per 100,000 patient-years. Unnatural deaths per admission have declined over time, but no significant change was observed in the analysis of deaths per person year.

Suicide accounted for 4.8% of all deaths, with a frequency of 5.4 deaths per 10,000 admissions and at a rate of 139.9 deaths per 100,000 patient-years. No significant temporal trends were observed in suicides per admission or rates per person year.

Accidental deaths comprised 3.3% of all deaths and occurred at a frequency of 6.5 deaths per 10,000 admissions, at a rate of 92.1 deaths per 100,000 patient-years. Accidental deaths declined over time as measured by deaths per admission, but not per person per year.

Homicides comprised 1.7% of all deaths and occurred at a frequency of 4.5 deaths per 10,000 admissions, at a rate of 2.4 deaths per 100,000 person-years. Only three of the 38 studies reported homicide deaths, although some homicides might have been included in unspecified deaths.

### Unknown and unspecified deaths

Fifteen studies reported a pooled estimate of 10.6% (95% CI, 6.8–16.1) of deaths with an unspecified cause. Twenty-two studies reported a pooled estimate of 13.8% (9.1–20.3) of deaths with an unknown cause. Seven studies reported both unspecified and unknown deaths. An analysis of the thirty studies that reported either unknown or unspecified deaths (including the total of the unknown and unspecified deaths in the seven studies that reported both) estimated that 15.2% (95% CI, 9.9–22.7) of deaths were not ascribed a specific cause. It is likely that some of the deaths with less common causes that were known to researchers were included in the unspecified group. Unknown causes likely included the results of fatal deliberate self-harm with a non-concluded or open coroner's verdict.

### Mortality associated with non-CODeM causes

Eleven papers referenced ‘sudden death’ as a cause of inpatient mortality, accounting for 14.1% of deaths at 7.8 deaths per 10,000 admissions, and a rate of 395 deaths per 100,000 patient years. Sudden deaths have remained stable over time. These deaths likely overlap with vascular and respiratory deaths.

Seven studies recorded cases of choking or asphyxia, accounting for 4.3% of deaths, at 2.1 deaths per 10,000 admissions, at a rate of 72.9 deaths per 100,000 person years.

Six studies recorded deaths from neuroleptic malignant syndrome (NMS), and four reported electroconvulsive therapy-related (ECT-related) deaths. No study specified deaths from lithium toxicity, clozapine, or hyponatraemia.

### Sensitivity analysis according to study nation income grouping and language

Studies performed in HI countries had a significantly higher proportion of deaths from unnatural causes. In contrast, the differences in proportions of total natural and cause-specific deaths were not statistically significant (Supplementary Table S5).

Studies from high-income countries reported a higher number of total natural, total unnatural and total deaths per 10,000 admissions. This was reflected in higher mortality per admission from accident, suicide, gastrointestinal, respiratory, and vascular causes (Supplementary Table S6).

Studies from HI countries had higher rates of unnatural deaths per 100,000 patient years than studies performed in LAMI settings. High-income countries had higher rates of respiratory, vascular and suicide deaths. LAMI countries had higher rates of infectious and metabolic deaths (Supplementary Table S7).

Use of English as a national language was associated with a lower proportion of mortalities that were not specified (Supplementary Table S9), a lower number of unspecified deaths and suicides per admission (SupplementaryTable S10), and a lower rate of deaths attributed to mental and behavioural conditions, not specified causes, and unknown causes (Supplementary Table S11).

### Heterogeneity and publication bias

I-square statistics were very high for every mortality, excepting homicide and ECT-related deaths, which were reported in too few studies for a meaningful heterogeneity assessment. Adjustments for publication bias when Egger's regression had a P < 0.05 can be found in Supplementary Tables S12–S14. Residual heterogeneity remained highly elevated for every mortality after meta-regression for publication date.

## Discussion

To our knowledge, this is the first meta-analysis of cause-specific mortalities among currently admitted psychiatric inpatients. The study estimated that 8 in 10 deaths in this setting are from natural causes, while about 1 in 20 are suicides. In descending order, the three leading causes of death per 100,000 patient-years were vascular, infectious, and respiratory deaths. Sudden natural death was more common than suicide, and choking deaths were only slightly less common than all accidents. The preponderance of natural mortality suggests that physical health causes of death may be an under-recognised and under-researched cause of inpatient mortality.

The study identified trends in psychiatric hospital deaths in the last 50 years. First, there seems to have been a dramatic decline in the number of inpatient deaths per 10,000 admissions. This statistic can be expressed as the expected number of psychiatric admissions per death; among papers published prior to 2000, about 1 in 28 admissions ended in death, while the equivalent figure in papers published after this date was 1 in 216. This fall was reflected in observed mortalities from vascular, infectious, respiratory, neoplastic, neurological, and accidental death. An overall reduction in deaths per admission in inpatient settings could result from reduced time at risk in the era of a short length of stay and deinstitutionalisation.^71^ However, the study also found an approximate 75% reduction in natural and total inpatient mortality, as measured by deaths per patient-year. Unlike deaths per admission, mortality rates per patient year are insensitive to length of stay and the fall in mortality rates is reassuring. This reduction in mortality was associated with fewer deaths from infectious, neoplastic, respiratory, and sudden causes.

The prominence of vascular deaths in inpatient settings is consistent with deaths in the community, as outlined in the Global Burden of Disease Study (GBD 2017)^72^ and with studies of mortality after psychiatric discharge.^15^^,^^16^ However, our rate of vascular mortality was more than four times that reported in the GBD study and 50% higher than the recent meta-analytic estimate among discharged psychiatric patients.^15^ While this study and the GBD 2017 used CODEm to classify deaths, in other respects, the methods are not directly comparable. However, raised inpatient vascular mortality is consistent with what is known about vascular risk among psychiatric patients and likely reflects the challenges of managing vascular risk factors among people with serious mental illness. Contributing to this complexity is the prevalence of multimorbidity and an earlier average age of onset of illness compared to the general population, as well as the impact of lifestyle-related risks, disorganisation commonly associated with severe mental illness, and the metabolic side effects of psychotropic medications.^2^

Our findings raise questions about an elevation of mortality associated with inpatient psychiatric care when compared to mortality after discharge.^15^ The overall rate of natural mortality among current psychiatric inpatients in our analysis was 2905 per 100,000 person-years, more than two and a half times higher than a recent estimate of post-discharge natural mortality among discharged patients of 1128 per 100,000 person-years reported in a 2019 meta-analysis by Swaraj and associates.^15^ The most increased mortality was infectious, with the estimate among inpatients being eight times higher than that derived from a comparable meta-analysis of discharged patients.^15^ Some of these apparent differences in mortality might be a result of methodological differences. While the present study and the Swaraj meta-analysis both examined hospitalised psychiatric patients, used similar data bases, employed the same analytic methods, and used the CODEm system, the studies differed in that the present meta-analysis sampled fewer primary studies (38 vs 71), included studies with a somewhat later median publication date (2005 vs 2001), and sampled a larger proportion of studies from LAMI countries (39% vs 1.4%). The presence of more studies from a LAMI setting might be the most important of these factors because LAMI studies in this meta-analysis reported infectious mortality that was about three times higher than studies in HI settings.

Measurement bias in primary studies may also have contributed to the high rate of infectious deaths among inpatients. Primary studies in both meta-analyses rarely reported the infectious pathogens causing deaths. It is possible that studies of inpatients and discharged patients tended to code infectious deaths differently. For example, cases of pneumonia or gastroenteritis might be coded respectively as respiratory or gastrointestinal deaths in the community but as infectious deaths in hospitals, particularly after an outbreak of an infectious illness.

Several factors might generally contribute to an elevated mortality observed among psychiatric inpatients. The severity of psychiatric illness among admitted patients might particularly impair patient's ability to communicate physical symptoms or staff's ability to recognise clinical deterioration. This sort of diagnostic overshadowing, where physical symptoms are misattributed to psychiatric conditions, is known to delay access to appropriate medical care^11^ and might be more marked in an inpatient setting. Additionally, adherence to physiological monitoring protocols, well-established in general hospital settings,^73^ may be more challenging to implement consistently in psychiatric units. As a result, physically unwell patients may sometimes only be identified when critically ill, increasing the risk of fatal outcomes.

It is possible that the psychosocial stress of a severe mental illness and hospitalisation experienced by inpatients confers direct risks to physical health. Psychological stress is known to be associated with both vascular illness^74^ and respiratory tract infections.^75^

It is also possible that a proportion of infectious inpatient deaths are quite conventionally nosocomial. The environmental and structural characteristics of psychiatric facilities might heighten vulnerability to the transmission of infectious disease. Inpatient psychiatric units are often enclosed, with shared facilities, high patient mobility, and limited access to small communal outdoor areas. Infection control measures such as isolation, mask-wearing, and hand hygiene may be challenging to enforce, particularly among patients with behavioural or cognitive impairments. Restrictions on access to hand sanitiser and sinks, implemented for suicide safety reasons, may further limit effective hygiene practices.^76^ Consistent with our findings, infection is a common reason for medical emergency calls^77^ and in one study, infection was the most common reason for transfer from a psychiatric to a medical setting.^78^

This study has notable limitations. The most notable limitation of the study is the very high between-study heterogeneity for almost all mortalities, irrespective of how they were measured. All meta-analyses are affected by the availability of primary literature, and the diverse settings, patient populations, and the differing decades of the studies likely contributed to the between-study heterogeneity. This high between-study heterogeneity, whilst partially explained by the moderator of publication date, limits the generalisability of our calculated mortalities. While the diverse range of national settings in which the research was performed reduced the risk of bias associated with geographic and language factors, this diversity almost certainly contributed to the high between study heterogeneity we observed, such that there might not be an expected rate of mortality from specific causes in psychiatric hospitals, but rather that the rates of death are dictated by a range of patient, health service, and demographic factors that have yet to be examined and reported in the literature. Another possible source of unexplained between study heterogeneity are differences in the demographic and diagnostic profiles in the primary study population. In this study too few studies reported the age, sex, and diagnostic mix of the patient population for a meaningful examination of these potentially important drivers of mortality.

The included primary studies also had some weaknesses. First, the included studies reported a mean of less than half of the CODeM mortalities, and many studies included a proportion of deaths from either unknown or unspecified causes. Had the cause of these deaths been known and reported, the estimates for specific causes might have differed. Second, the incomplete set of mortality causes in many studies reduced the number of data points for every mortality and raises the possibility that some mortalities were reported because of a high number of deaths. This publication bias at the level of mortality would have the effect of inflating the mortalities of more common specific causes, because studies with low numbers of death from common causes might have been coded as unspecified. The use of an unspecified category might also have concealed the magnitude of some less common mortalities. Third, there were probable inconsistencies in the terminology used to classify causes of death because some studies did not utilise an external coding system. In the absence of a standardised external coding system, such classifications may have varied between studies, potentially introducing inconsistencies in categorisation, for example delirium tremens might have been recorded inconsistently in either the mental and behavioural disorders or neurological category. Fourth, the total number of admissions and patient years was not always reported, leading to the exclusion of some studies from estimates of the observed mortality per admission and mortality rates. Finally, the primary studies did not report sufficient information to meta-analyse the effect of the important community drivers of mortality, such as age and sex.

With these limitations and the degree of unexplained heterogeneity in mind, the elevated mortality rates among inpatients highlight the need for integrated physical and mental health care in psychiatric hospitals. The provision of better physical health care in these settings must include; i) measures to ascertain the diagnoses and treatment of existing conditions, including by improved medication reconciliation^79^ and communication between mental health and primary care providers,^80^ ii) improved health screening, diagnosis, and management of modifiable health conditions, most importantly of cardiometabolic risk factors,^81^ iii) measures to ensure the recognition and management of clinical deterioration^73^^,^^82^ and iv) ways of ensuring that people with significant medical and psychiatric comorbidity receive optimal care for life-threatening conditions in the most appropriate setting.^83^

Our findings also emphasise the need to improve infection control in inpatient psychiatric settings. This is likely to require improved screening for infectious diseases,^84^ higher vaccination rates,^85^ changes in ward procedures,^86^ including better systems for detection of outbreaks, and ultimately improved ward design.

Future initiatives should continue to build on existing knowledge about the integration of physical health and psychiatric care^87^ while refining strategies to enhance patient engagement and support for improved health outcomes. Future reductions in inpatient and post discharge mortality will require a determined focus on the health needs of people hospitalised with mental illness, such that mental health services become health services for people with mental illness.

## Contributors

Conceptualisation: PB, JC, PG, ML, GS.

Accessed and verified the data: AB, HA-S, AT, ML.

Data Analysis: AT, HA-S, ML, Data Interpretation: PB, JC, PG, GS.

Methodology: AB, HA-S, PB, ML, GS.

Project Supervision: ML.

Funding Acquisition: Unfunded.

Writing—Original Draft: HA-S.

Writing—Review and Editing: AB, HA-S, AT, PB, JC, PG, ML, GS.

## Data sharing statement

The extracted data used in the meta-analyses can be found in Supplementary Material 8. Data used in meta-analysis of cause specific mortality in psychiatric hospitals.

## Declaration of interests

ML has provided evidence in coroners matters after deaths in psychiatric hospitals, HAS received speakers Honoraria for Gilead Beyond Undetectable 2024, AB is a Board Director of the Royal Australian and New Zealand College of Psychiatrists. AT, PG, PB, GS, and JC have no conflicts of interest.

## Appendix A Supplementary data

Supplementary Tables

Supplementary Table and Data used in meta-analysis
