# Supplementary material for: Natural, unnatural, and cause-specific mortality among current psychiatric inpatients: a systematic review and meta-analysis
Source: eClinicalMedicine. 2025 Nov 14;90:103635. doi: 10.1016/j.eclinm.2025.103635 (PMC12664350; doi:10.1016/j.eclinm.2025.103635)
Supplement: Supplementary Tables [file mmc1.docx]

| **Supplementary Material 1. Strength of Reporting** | | | | | | |
| --- | --- | --- | --- | --- | --- | --- |
| **Paper** | **Cause of death classified using a recognised diagnostic system** | **Deaths ascertained using an external mortality database** | **Completeness of mortality**  **> 85%** | **Defined Catchment area of patients** | **Representative - not selected by age/diagnosis** | **Sum** |
| **Aberra 2018** | 0 | 0 | 0 | 1 | 1 | 2 |
| **Abiodun 1988** | 0 | 0 | 0 | 0 | 1 | 1 |
| **Adelugba 2018** | 0 | 0 | 1 | 0 | 1 | 2 |
| **Ali 2021** | 0 | 0 | 0 | 1 | 1 | 2 |
| **Ballinger 1976** | 0 | 0 | 1 | 1 | 1 | 3 |
| **Barbosa 2016** | 1 | 0 | 0 | 1 | 1 | 3 |
| **Black 1992** | 1 | 1 | 1 | 1 | 1 | 5 |
| **Brook 1985** | 0 | 1 | 0 | 1 | 1 | 3 |
| **Casadebaig 1989** | 1 | 1 | 1 | 1 | 1 | 5 |
| **Cengisiz 2023** | 1 | 0 | 0 | 0 | 1 | 2 |
| **Colton 2006** | 1 | 0 | 0 | 1 | 1 | 3 |
| **Costa 1981** | 0 | 0 | 1 | 1 | 1 | 3 |
| **Craig 1983** | 1 | 0 | 1 | 0 | 1 | 3 |
| **Craig 1984** | 1 | 0 | 1 | 0 | 1 | 3 |
| **Ferriter 2016** | 1 | 1 | 1 | 0 | 1 | 4 |
| **Fulga 2021** | 0 | 1 | 1 | 0 | 1 | 3 |
| **Giel 1978** | 0 | 1 | 0 | 1 | 1 | 3 |
| **Gunaratne 2023** | 1 | 1 | 1 | 1 | 1 | 5 |
| **Hewer 1995** | 0 | 0 | 0 | 1 | 1 | 2 |
| **Hwang 1998** | 0 | 0 | 1 | 0 | 0 | 1 |
| **Ifteni 2014** | 0 | 0 | 1 | 0 | 0 | 1 |
| **Kaggwa 2021** | 0 | 0 | 0 | 0 | 1 | 1 |
| **Kamara 1998** | 1 | 0 | 1 | 0 | 1 | 3 |
| **Khamker 2010** | 0 | 0 | 1 | 0 | 1 | 2 |
| **Khlil 2023** | 0 | 0 | 1 | 1 | 1 | 3 |
| **Lim 1991** | 0 | 0 | 1 | 1 | 1 | 3 |
| **Malomo 2003** | 1 | 0 | 1 | 1 | 1 | 4 |
| **Ndosi 1997** | 0 | 0 | 0 | 0 | 1 | 1 |
| **Ojansuu 2018** | 0 | 1 | 1 | 1 | 1 | 4 |
| **Osman 2018** | 0 | 0 | 0 | 1 | 1 | 2 |
| **Osman 2020** | 0 | 0 | 0 | 1 | 1 | 2 |
| **Pérez-Cárceles 2001** | 1 | 0 | 1 | 1 | 1 | 4 |
| **Saito 2014** | 0 | 0 | 1 | 0 | 0 | 1 |
| **Saugstad 1979** | 0 | 1 | 1 | 1 | 1 | 4 |
| **Shinde 2014** | 0 | 0 | 1 | 0 | 1 | 2 |
| **Shinozaki 1976** | 1 | 1 | 1 | 1 | 1 | 5 |
| **Swain 2019** | 0 | 0 | 1 | 0 | 1 | 2 |
| **Wu 2022** | 0 | 0 | 0 | 1 | 1 | 2 |

| SM 2. Proportions of inpatient deaths according to cause in studies before and after 2000 | | | | | | | | |
| --- | --- | --- | --- | --- | --- | --- | --- | --- |
|  |  | n. studies | Estimate | Lower limit | Upper limit | Q-value | df (q) | P-value |
| Gastrointestinal | <2000 | 5 | 3% | 2% | 5% | 3.15 | 1.00 | 0.08 |
|  | >2000 | 9 | 7% | 3% | 14% |  |  |  |
| Infectious | <2000 | 12 | 18% | 10% | 31% | 0.06 | 1.00 | 0.81 |
|  | >2000 | 10 | 17% | 10% | 28% |  |  |  |
| Ment. & Beh. | <2000 | 2 | 3% | 1% | 8% | 0.41 | 1.00 | 0.52 |
|  | >2000 | 4 | 4% | 2% | 9% |  |  |  |
| Metabolic | <2000 | 7 | 4% | 2% | 9% | 0.00 | 1.00 | 0.99 |
|  | >2000 | 10 | 4% | 2% | 7% |  |  |  |
| Neoplastic | <2000 | 15 | 7% | 6% | 8% | 0.20 | 1.00 | 0.66 |
|  | >2000 | 7 | 6% | 4% | 10% |  |  |  |
| Neurological | <2000 | 6 | 4% | 2% | 8% | 4.90 | 1.00 | 0.03 |
|  | >2000 | 11 | 9% | 7% | 11% |  |  |  |
| Respiratory | <2000 | 9 | 18% | 11% | 27% | 1.11 | 1.00 | 0.29 |
|  | >2000 | 15 | 12% | 6% | 21% |  |  |  |
| Vascular | <2000 | 16 | 29% | 24% | 35% | 0.96 | 1.00 | 0.33 |
|  | >2000 | 19 | 33% | 27% | 40% |  |  |  |
| Not Specified | <2000 | 8 | 8% | 5% | 13% | 1.42 | 1.00 | 0.23 |
|  | >2000 | 7 | 14% | 6% | 31% |  |  |  |
| Total Natural | <2000 | 15 | 85% | 76% | 90% | 1.19 | 1.00 | 0.28 |
|  | >2000 | 22 | 79% | 71% | 85% |  |  |  |
| Accident | <2000 | 10 | 3% | 2% | 5% | 0.22 | 1.00 | 0.64 |
|  | >2000 | 9 | 4% | 2% | 6% |  |  |  |
| Homicide | <2000 | 1 | 1% | 0% | 6% | 0.69 | 1.00 | 0.41 |
|  | >2000 | 2 | 2% | 1% | 6% |  |  |  |
| Suicide | <2000 | 9 | 3% | 2% | 5% | 2.11 | 1.00 | 0.15 |
|  | >2000 | 16 | 6% | 3% | 12% |  |  |  |
| Total Unnatural | <2000 | 15 | 6% | 5% | 7% | 0.58 | 1.00 | 0.45 |
|  | >2000 | 22 | 7% | 4% | 12% |  |  |  |
| Unknown | <2000 | 8 | 14% | 7% | 25% | 0.00 | 1.00 | 0.98 |
|  | >2000 | 14 | 14% | 7% | 24% |  |  |  |
| Sudden | <2000 | 3 | 14% | 9% | 22% | 0.02 | 1.00 | 0.88 |
|  | >2000 | 8 | 13% | 6% | 25% |  |  |  |
| Choking | <2000 | 2 | 2% | 0% | 11% | 0.86 | 1.00 | 0.35 |
|  | >2000 | 5 | 6% | 2% | 17% |  |  |  |
| ECT | <2000 | 1 | 1% | 0% | 5% | 1.35 | 1.00 | 0.25 |
|  | >2000 | 3 | 3% | 1% | 7% |  |  |  |
| NMS | <2000 | 1 | 6% | 1% | 31% | 0.01 | 1.00 | 0.91 |
|  | >2000 | 5 | 6% | 2% | 16% |  |  |  |

| SM 3. Inpatient deaths per 10,000 admissions according to cause in studies before and after 2000 | | | | | | | | |
| --- | --- | --- | --- | --- | --- | --- | --- | --- |
|  |  | n. studies | Estimate | Lower limit | Upper limit | Q-value | df (q) | P-value |
| Gastrointestinal | <2000 | 4 | 8.9 | 6.6 | 12.0 | 2.47 | 1 | 0.12 |
|  | >2000 | 8 | 3.3 | 1.0 | 11.0 |  |  |  |
| Infectious | <2000 | 8 | 69.5 | 30.3 | 158.8 | 5.70 | 1 | 0.02 |
|  | >2000 | 7 | 14.7 | 5.6 | 38.7 |  |  |  |
| Ment. & Beh. | <2000 | 2 | 6.6 | 0.9 | 47.9 | 3.36 | 1 | 0.07 |
|  | >2000 | 3 | 1.0 | 0.8 | 1.3 |  |  |  |
| Metabolic | <2000 | 6 | 8.2 | 4.3 | 15.3 | 5.05 | 1 | 0.02 |
|  | >2000 | 7 | 2.0 | 0.7 | 5.7 |  |  |  |
| Neoplastic | <2000 | 11 | 22.4 | 13.4 | 37.1 | 4.40 | 1 | 0.04 |
|  | >2000 | 3 | 2.2 | 0.3 | 18.0 |  |  |  |
| Neurological | <2000 | 6 | 6.8 | 3.7 | 12.4 | 4.43 | 1 | 0.04 |
|  | >2000 | 10 | 2.7 | 1.4 | 5.0 |  |  |  |
| Respiratory | <2000 | 7 | 46.8 | 35.8 | 61.1 | 34.97 | 1 | <.0001 |
|  | >2000 | 9 | 4.5 | 2.2 | 9.3 |  |  |  |
| Vascular | <2000 | 12 | 77.6 | 42.4 | 141.6 | 23.88 | 1 | <.0001 |
|  | >2000 | 14 | 10.4 | 6.1 | 17.7 |  |  |  |
| Not Specified | <2000 | 5 | 14.5 | 2.1 | 96.9 | 1.31 | 1 | 0.25 |
|  | >2000 | 5 | 3.9 | 1.1 | 13.1 |  |  |  |
| Total Natural | <2000 | 11 | 246.6 | 132.9 | 453.2 | 18.77 | 1 | <.0001 |
|  | >2000 | 16 | 35.2 | 18.8 | 65.9 |  |  |  |
| Accident | <2000 | 8 | 12.2 | 6.0 | 24.8 | 5.49 | 1 | 0.02 |
|  | >2000 | 6 | 2.8 | 1.0 | 7.7 |  |  |  |
| Homicide | <2000 | 1 | 2.5 | 0.4 | 17.8 | 0.67 | 1 | 0.41 |
|  | >2000 | 1 | 8.0 | 1.1 | 56.4 |  |  |  |
| Suicide | <2000 | 8 | 14.9 | 11.4 | 19.4 | 5.59 | 1 | 0.02 |
|  | >2000 | 12 | 2.7 | 0.7 | 10.8 |  |  |  |
| Total Unnatural | <2000 | 11 | 27.3 | 17.9 | 41.7 | 14.69 | 1 | 0.0001 |
|  | >2000 | 16 | 2.9 | 1.0 | 8.4 |  |  |  |
| Unknown | <2000 | 6 | 70.8 | 25.5 | 194.9 | 27.49 | 1 | <.0001 |
|  | >2000 | 10 | 2.9 | 1.6 | 5.4 |  |  |  |
| Sudden | <2000 | 2 | 22.0 | 15.6 | 31.1 | 10.30 | 1 | 0.001 |
|  | >2000 | 7 | 5.3 | 2.4 | 11.8 |  |  |  |
| Choking | <2000 | 2 | 4.8 | 0.5 | 43.6 | 0.76 | 1 | 0.38 |
|  | >2000 | 4 | 1.5 | 0.3 | 6.4 |  |  |  |
| ECT | <2000 | 1 | 0.9 | 0.1 | 6.7 | 0.70 | 1 | 0.40 |
|  | >2000 | 3 | 2.4 | 0.8 | 7.1 |  |  |  |
| NMS | <2000 | 1 | 8.3 | 1.2 | 58.5 | 1.12 | 1 | 0.29 |
|  | >2000 | 5 | 2.7 | 1.3 | 5.4 |  |  |  |
| Total death | <2000 | 11 | 359.7 | 246.2 | 522.7 | 28.44 | 1 | <.0001 |
|  | >2000 | 15 | 46.4 | 24.1 | 89.1 |  |  |  |

| SM 4. Inpatient deaths per 100,000 person years according to cause in studies before and after 2000 | | | | | | | | |
| --- | --- | --- | --- | --- | --- | --- | --- | --- |
|  |  | n. studies | Estimate | Lower limit | Upper limit | Q-value | df (q) | P-value |
| Gastrointestinal | <2000 | 4 | 168.4 | 115.6 | 221.2 | 5.88 | 1 | 0.015 |
|  | >2000 | 7 | 74.9 | 20.7 | 129.0 |  |  |  |
| Infectious | <2000 | 9 | 902.3 | 663.6 | 1140.9 | 9.34 | 1 | 0.002 |
|  | >2000 | 6 | 423.7 | 230.7 | 616.7 |  |  |  |
| Mental & Beh. | <2000 | 2 | 163.1 | -95.9 | 422.2 | 0.01 | 1 | 0.924 |
|  | >2000 | 2 | 183.9 | -159.5 | 527.4 |  |  |  |
| Metabolic | <2000 | 6 | 244.5 | 80.1 | 409.0 | 7.98 | 1 | 0.005 |
|  | >2000 | 5 | 6.8 | 0.0 | 19.0 |  |  |  |
| Neoplastic | <2000 | 10 | 275.7 | 202.1 | 349.4 | 17.71 | 1 | <.0001 |
|  | >2000 | 6 | 85.2 | 35.7 | 134.7 |  |  |  |
| Neurological | <2000 | 6 | 105.1 | 6.2 | 204.1 | 1.09 | 1 | 0.30 |
|  | >2000 | 7 | 175.5 | 88.3 | 262.6 |  |  |  |
| Respiratory | <2000 | 7 | 1304.0 | 782.4 | 1825.7 | 19.82 | 1 | <.0001 |
|  | >2000 | 8 | 114.4 | 67.6 | 161.2 |  |  |  |
| Vascular | <2000 | 11 | 1466.2 | 1053.6 | 1878.9 | 16.41 | 1 | <.0001 |
|  | >2000 | 12 | 534.7 | 353.4 | 716.0 |  |  |  |
| Not Specified | <2000 | 7 | 607.8 | 225.2 | 990.3 | 0.42 | 1 | 0.52 |
|  | >2000 | 5 | 452.7 | 182.9 | 722.4 |  |  |  |
| Total Natural | <2000 | 11 | 5445.4 | 4460.9 | 6429.9 | 56.14 | 1 | <.0001 |
|  | >2000 | 14 | 1374.3 | 968.3 | 1780.2 |  |  |  |
| Accident | <2000 | 7 | 116.0 | 70.3 | 161.6 | 4.92 | 1 | 0.03 |
|  | >2000 | 5 | 43.7 | 0.0 | 88.3 |  |  |  |
| Homicide | <2000 | 1 | 21.7 | 0.0 | 64.3 | 0.80 | 1 | 0.37 |
|  | >2000 | 2 | 2.3 | 0.0 | 5.3 |  |  |  |
| Suicide | <2000 | 7 | 167.8 | 87.1 | 248.4 | 0.64 | 1 | 0.42 |
|  | >2000 | 10 | 129.3 | 80.6 | 178.1 |  |  |  |
| Total Unnatural | <2000 | 10 | 272.2 | 152.5 | 392.0 | 3.39 | 1 | 0.07 |
|  | >2000 | 14 | 150.3 | 100.6 | 200.1 |  |  |  |
| Unknown | <2000 | 5 | 1664.4 | 1114.8 | 2214.0 | 25.93 | 1 | <.0001 |
|  | >2000 | 8 | 205.3 | 89.5 | 321.1 |  |  |  |
| Sudden | <2000 | 3 | 868.0 | 247.6 | 1488.3 | 3.92 | 1 | 0.048 |
|  | >2000 | 5 | 219.4 | 55.3 | 383.5 |  |  |  |
| Choking | <2000 | 2 | 113.4 | 19.1 | 207.7 | 0.45 | 1 | 0.50 |
|  | >2000 | 3 | 73.2 | 3.1 | 143.3 |  |  |  |
| ECT | <2000 | 1 | 14.3 | 0.0 | 42.5 | 0.38 | 1 | 0.54 |
|  | >2000 | 1 | 41.4 | 0.0 | 122.6 |  |  |  |
| NMS | <2000 | 1 | 1136.4 | 0.0 | 3363.6 | 0.94 | 1 | 0.33 |
|  | >2000 | 3 | 32.6 | 0.0 | 84.8 |  |  |  |
| Total death | <2000 | 10 | 7955.1 | 6391.8 | 9518.3 | 53.09 | 1 | <.0001 |
|  | >2000 | 14 | 1806.8 | 1267.1 | 2346.5 |  |  |  |

| SM 5. Proportions of inpatient deaths according to cause in studies by country income status | | | | | | | | |
| --- | --- | --- | --- | --- | --- | --- | --- | --- |
|  |  | n. studies | Estimate | Lower limit | Upper limit | Q-value | df (q) | P-value |
| Gastrointestinal | LAMI | 7 | 3.5% | 2.2% | 5.7% | 3.22 | 1 | 0.07 |
|  | HI | 7 | 8.6% | 3.7% | 18.8% |  |  |  |
| Infectious | LAMI | 9 | 23.8% | 11.3% | 43.4% | 1.37 | 1 | 0.24 |
|  | HI | 13 | 14.0% | 8.1% | 23.2% |  |  |  |
| Ment. and Beh. | LAMI | 2 | 3.3% | 1.9% | 5.6% | 0.07 | 1 | 0.79 |
|  | HI | 4 | 3.7% | 1.8% | 7.6% |  |  |  |
| Metabolic | LAMI | 10 | 3.9% | 2.0% | 7.5% | 0.12 | 1 | 0.73 |
|  | HI | 7 | 3.2% | 1.2% | 8.2% |  |  |  |
| Neoplastic | LAMI | 5 | 4.6% | 2.8% | 7.6% | 2.49 | 1 | 0.11 |
|  | HI | 17 | 7.1% | 5.9% | 8.5% |  |  |  |
| Neurological | LAMI | 10 | 7.0% | 5.0% | 9.8% | 0.18 | 1 | 0.67 |
|  | HI | 7 | 6.2% | 3.7% | 10.0% |  |  |  |
| Respiratory | LAMI | 9 | 16.2% | 9.3% | 26.7% | 0.37 | 1 | 0.55 |
|  | HI | 15 | 13.3% | 9.3% | 18.7% |  |  |  |
| Vascular | LAMI | 14 | 28.9% | 22.5% | 36.1% | 0.56 | 1 | 0.45 |
|  | HI | 21 | 32.1% | 27.2% | 37.5% |  |  |  |
| Not Specified | LAMI | 6 | 15.1% | 3.9% | 43.8% | 0.37 | 1 | 0.54 |
|  | HI | 9 | 9.9% | 6.9% | 14.0% |  |  |  |
| Total Natural | LAMI | 15 | 83.9% | 72.1% | 91.3% | 0.56 | 1 | 0.45 |
|  | HI | 22 | 79.3% | 71.8% | 85.2% |  |  |  |
| Accident | LAMI | 7 | 3.9% | 2.3% | 6.6% | 0.54 | 1 | 0.46 |
|  | HI | 12 | 3.0% | 1.9% | 4.8% |  |  |  |
| Homicide | LAMI |  |  |  |  |  |  |  |
|  | HI | 3 | 1.7% | 0.6% | 4.4% |  |  |  |
| Suicide | LAMI | 10 | 3.8% | 2.3% | 6.4% | 0.90 | 1 | 0.34 |
|  | HI | 15 | 5.7% | 3.0% | 10.3% |  |  |  |
| Total Unnatural | LAMI | 15 | 4.1% | 2.4% | 7.0% | 6.43 | 1 | 0.01 |
|  | HI | 22 | 9.2% | 6.7% | 12.5% |  |  |  |
| Unknown | LAMI | 9 | 18.6% | 9.5% | 33.0% | 1.41 | 1 | 0.23 |
|  | HI | 13 | 11.2% | 6.4% | 18.8% |  |  |  |
| Sudden | LAMI | 9 | 14.4% | 8.1% | 24.1% | 0.34 | 1 | 0.56 |
|  | HI | 2 | 11.6% | 7.0% | 18.4% |  |  |  |
| Choking | LAMI | 4 | 2.1% | 0.6% | 6.8% | 3.06 | 1 | 0.08 |
|  | HI | 3 | 9.0% | 2.9% | 24.3% |  |  |  |
| ECT | LAMI | 4 | 2.0% | 0.8% | 4.8% |  |  |  |
|  | HI |  |  |  |  |  |  |  |
| NMS | LAMI | 5 | 6.3% | 2.4% | 15.6% | 0.01 | 1 | 0.91 |
|  | HI | 1 | 5.6% | 0.8% | 30.7% |  |  |  |

| SM 6. Inpatient deaths per 10,000 admissions according to cause in studies by country income status | | | | | | | | |
| --- | --- | --- | --- | --- | --- | --- | --- | --- |
|  |  | n. studies | Estimate | Lower limit | Upper limit | Q-value | df (q) | P-value |
| Gastrointestinal | LAMI | 7 | 1.7 | 0.7 | 4.4 | 14.23 | 1 | 0.0002 |
|  | HI | 5 | 14.4 | 8.0 | 26.0 |  |  |  |
| Infectious | LAMI | 7 | 22.7 | 8.8 | 58.4 | 1.24 | 1 | 0.2649 |
|  | HI | 8 | 50.1 | 18.1 | 138.5 |  |  |  |
| Ment & Beh. | LAMI | 2 | 1.1 | 0.6 | 1.9 | 0.91 | 1 | 0.3391 |
|  | HI | 3 | 3.3 | 0.4 | 30.1 |  |  |  |
| Metabolic | LAMI | 8 | 2.6 | 0.9 | 7.6 | 2.54 | 1 | 0.1109 |
|  | HI | 5 | 7.3 | 3.7 | 14.6 |  |  |  |
| Neoplastic | LAMI | 3 | 3.6 | 0.8 | 17.2 | 3.24 | 1 | 0.0717 |
|  | HI | 11 | 17.4 | 8.7 | 34.5 |  |  |  |
| Neurological | LAMI | 10 | 3.5 | 2.1 | 6.1 | 0.02 | 1 | 0.8841 |
|  | HI | 6 | 3.2 | 0.9 | 11.3 |  |  |  |
| Respiratory | LAMI | 6 | 4.0 | 2.1 | 7.8 | 12.65 | 1 | 0.0004 |
|  | HI | 10 | 23.0 | 11.5 | 46.0 |  |  |  |
| Vascular | LAMI | 12 | 10.2 | 6.2 | 16.7 | 16.01 | 1 | 0.0001 |
|  | HI | 14 | 57.0 | 28.8 | 112.2 |  |  |  |
| Not Specified | LAMI | 4 | 4.8 | 1.3 | 17.5 | 0.26 | 1 | 0.6129 |
|  | HI | 6 | 9.8 | 0.9 | 110.3 |  |  |  |
| Total Natural | LAMI | 12 | 32.4 | 17.1 | 61.4 | 10.16 | 1 | 0.0014 |
|  | HI | 15 | 161.1 | 76.1 | 338.0 |  |  |  |
| Accident | LAMI | 5 | 2.1 | 0.8 | 5.8 | 8.19 | 1 | 0.0042 |
|  | HI | 9 | 12.5 | 6.3 | 24.5 |  |  |  |
| Homicide | LAMI |  |  |  |  |  |  |  |
|  | HI | 2 | 4.5 | 1.1 | 17.9 |  |  |  |
| Suicide | LAMI | 9 | 1.7 | 0.9 | 3.2 | 16.38 | 1 | 0.0001 |
|  | HI | 11 | 12.7 | 6.0 | 26.7 |  |  |  |
| Total Unnatural | LAMI | 12 | 2.1 | 1.1 | 4.1 | 24.67 | 1 | <.0001 |
|  | HI | 15 | 21.7 | 11.4 | 41.1 |  |  |  |
| Unknown | LAMI | 8 | 4.6 | 2.3 | 9.1 | 6.37 | 1 | 0.0116 |
|  | HI | 8 | 22.1 | 8.1 | 60.6 |  |  |  |
| Sudden | LAMI | 8 | 6.7 | 3.3 | 13.7 | 4.71 | 1 | 0.0300 |
|  | HI | 1 | 17.6 | 10.6 | 29.3 |  |  |  |
| Choking | LAMI | 4 | 0.9 | 0.3 | 3.3 | 8.62 | 1 | 0.0033 |
|  | HI | 2 | 8.6 | 3.9 | 19.1 |  |  |  |
| ECT | LAMI | 4 | 2.1 | 0.8 | 5.5 |  |  |  |
|  | HI |  |  |  |  |  |  |  |
| NMS | LAMI | 5 | 2.7 | 1.3 | 5.4 | 1.12 | 1 | 0.2894 |
|  | HI | 1 | 8.3 | 1.2 | 58.5 |  |  |  |
| Total death | LAMI | 11 | 42.9 | 22.4 | 82.1 | 15.26 | 1 | 0.0001 |
|  | HI | 15 | 226.2 | 133.9 | 379.4 |  |  |  |

| SM 7. Inpatient deaths per 100,000 person years according to cause in studies by country income status | | | | | | | | |
| --- | --- | --- | --- | --- | --- | --- | --- | --- |
|  |  | n. studies | Estimate | Lower limit | Upper limit | Q-value | df (q) | P-value |
| Gastrointestinal | LAMI | 4 | 113.8 | 22.4 | 205.2 | 0.00 | 1 | 0.95 |
|  | HI | 7 | 110.4 | 39.0 | 181.8 |  |  |  |
| Infectious | LAMI | 4 | 1567.8 | 598.5 | 2537.1 | 4.94 | 1 | 0.03 |
|  | HI | 11 | 458.1 | 327.2 | 589.1 |  |  |  |
| Ment. and Beh. | LAMI | 4 | 166.8 | 37.7 | 296.0 |  |  |  |
|  | HI |  |  |  |  |  |  |  |
| Metabolic | LAMI | 4 | 175.1 | 98.9 | 251.3 | 15.71 | 1 | 0.0001 |
|  | HI | 7 | 17.1 | 0.0 | 34.5 |  |  |  |
| Neoplastic | LAMI | 3 | 165.1 | 0.0 | 406.2 | 0.11 | 1 | 0.74 |
|  | HI | 13 | 207.3 | 136.0 | 278.5 |  |  |  |
| Neurological | LAMI | 6 | 160.8 | 48.3 | 273.4 | 0.02 | 1 | 0.88 |
|  | HI | 7 | 149.9 | 60.2 | 239.7 |  |  |  |
| Respiratory | LAMI | 2 | 239.5 | 6.4 | 472.5 | 2.13 | 1 | 0.14 |
|  | HI | 13 | 426.9 | 332.4 | 521.4 |  |  |  |
| Vascular | LAMI | 7 | 525.5 | 328.1 | 722.9 | 13.26 | 1 | 0.0003 |
|  | HI | 16 | 1168.4 | 884.2 | 1452.7 |  |  |  |
| Not Specified | LAMI | 3 | 653.0 | 13.3 | 1292.8 | 0.12 | 1 | 0.7268 |
|  | HI | 9 | 529.7 | 266.3 | 793.0 |  |  |  |
| Total Natural | LAMI | 7 | 2287.3 | 1357.2 | 3217.4 | 1.87 | 1 | 0.171 |
|  | HI | 18 | 3078.0 | 2431.5 | 3724.5 |  |  |  |
| Accident | LAMI | 3 | 251.8 | 0.0 | 643.6 | 0.65 | 1 | 0.421 |
|  | HI | 9 | 89.4 | 38.0 | 140.9 |  |  |  |
| Homicide | LAMI |  |  |  |  |  |  |  |
|  | HI | 3 | 2.4 | 0.0 | 5.4 |  |  |  |
| Suicide | LAMI | 5 | 69.2 | 9.1 | 129.3 | 6.70 | 1 | 0.01 |
|  | HI | 12 | 172.3 | 122.5 | 222.1 |  |  |  |
| Total Unnatural | LAMI | 7 | 81.7 | 13.6 | 149.8 | 10.32 | 1 | 0.0013 |
|  | HI | 17 | 228.5 | 170.3 | 286.6 |  |  |  |
| Unknown | LAMI | 4 | 345.6 | 0.0 | 758.6 | 3.19 | 1 | 0.07 |
|  | HI | 9 | 833.5 | 492.4 | 1174.5 |  |  |  |
| Sudden | LAMI | 6 | 258.6 | 91.9 | 425.4 | 17.49 | 1 | <.0001 |
|  | HI | 2 | 1150.3 | 767.2 | 1533.5 |  |  |  |
| Choking | LAMI | 3 | 50.1 | 0.0 | 105.3 | 1.93 | 1 | 0.16 |
|  | HI | 2 | 117.2 | 40.3 | 194.2 |  |  |  |
| ECT | LAMI | 2 | 17.2 | 0.0 | 43.8 |  |  |  |
|  | HI |  |  |  |  |  |  |  |
| NMS | LAMI | 3 | 32.6 | 0.0 | 84.8 | 0.94 | 1 | 0.33 |
|  | HI | 1 | 1136.4 | 0.0 | 3363.6 |  |  |  |
| Total death | LAMI | 7 | 2971.1 | 1867.2 | 4075.0 | 2.30 | 1 | 0.13 |
|  | HI | 17 | 4092.8 | 3153.5 | 5032.1 |  |  |  |

| SM 9. Proportions of inpatient deaths according to English language | | | | | | | | |
| --- | --- | --- | --- | --- | --- | --- | --- | --- |
|  |  | n. studies | Estimate | Lower limit | Upper limit | Q-value | df (q) | P-value |
| Gastrointestinal | Non-English | 6.00 | 4.5% | 2.5% | 7.7% | 0.13 | 1.00 | 0.72 |
|  | English | 8.00 | 5.2% | 2.6% | 10.2% |  |  |  |
| Infectious | Non-English | 9.00 | 11.3% | 4.4% | 25.9% | 2.39 | 1.00 | 0.12 |
|  | English | 13.00 | 23.9% | 15.4% | 35.1% |  |  |  |
| Mental and beh | Non-English | 2.00 | 3.9% | 3.0% | 5.1% | 0.14 | 1.00 | 0.07 |
|  | English | 4.00 | 3.3% | 1.3% | 8.1% |  |  |  |
| Metabolic | Non-English | 7.00 | 3.5% | 1.4% | 8.5% | 0.08 | 1.00 | 0.77 |
|  | English | 10.00 | 4.1% | 2.3% | 7.1% |  |  |  |
| Neoplastic | Non-English | 10.00 | 5.5% | 4.4% | 6.8% | 4.65 | 1.00 | 0.03 |
|  | English | 12.00 | 8.0% | 6.1% | 10.3% |  |  |  |
| Neurological | Non-English | 8.00 | 6.5% | 3.4% | 12.2% | 0.01 | 1.00 | 0.91 |
|  | English | 9.00 | 6.8% | 5.1% | 9.0% |  |  |  |
| Respiratory | Non-English | 13.00 | 12.6% | 7.6% | 20.3% | 0.29 | 1.00 | 0.59 |
|  | English | 11.00 | 15.6% | 8.5% | 26.9% |  |  |  |
| Vascular | Non-English | 18.00 | 33.5% | 29.1% | 38.2% | 2.19 | 1.00 | 0.14 |
|  | English | 17.00 | 27.2% | 21.0% | 34.4% |  |  |  |
| Not Specified | Non-English | 7.00 | 22.1% | 13.1% | 34.7% | 14.48 | 1.00 | <.001 |
|  | English | 8.00 | 4.4% | 2.2% | 8.4% |  |  |  |
| Total Natural | Non-English | 20.00 | 76.8% | 68.5% | 83.4% | 2.98 | 1.00 | 0.08 |
|  | English | 17.00 | 84.7% | 78.7% | 89.2% |  |  |  |
| Accident | Non-English | 12.00 | 3.3% | 2.1% | 5.2% | 0.05 | 1.00 | 0.82 |
|  | English | 7.00 | 3.6% | 2.1% | 6.1% |  |  |  |
| Homicide | Non-English | 2.00 | 2.2% | 0.7% | 6.5% | 0.69 | 1.00 | 0.40 |
|  | English | 1.00 | 0.8% | 0.1% | 5.7% |  |  |  |
| Suicide | Non-English | 16.00 | 5.1% | 2.9% | 8.9% | 0.04 | 1.00 | 0.85 |
|  | English | 9.00 | 4.7% | 2.4% | 9.0% |  |  |  |
| Total Unnatural | Non-English | 21.00 | 7.5% | 5.4% | 10.2% | 0.46 | 1.00 | 0.50 |
|  | English | 16.00 | 6.2% | 3.9% | 9.7% |  |  |  |
| Unknown | Non-English | 12.00 | 17.8% | 10.5% | 28.5% | 1.60 | 1.00 | 0.21 |
|  | English | 10.00 | 9.9% | 4.5% | 20.5% |  |  |  |
| Sudden | Non-English | 6.00 | 18.7% | 8.6% | 35.7% | 1.61 | 1.00 | 0.20 |
|  | English | 5.00 | 10.2% | 5.7% | 17.5% |  |  |  |
| Choking | Non-English | 4.00 | 8.1% | 2.7% | 21.7% | 3.37 | 1.00 | 0.07 |
|  | English | 3.00 | 1.9% | 0.6% | 5.6% |  |  |  |
| ECT | Non-English | 3.00 | 2.6% | 1.0% | 6.8% | 1.35 | 1.00 | 0.25 |
|  | English | 1.00 | 0.7% | 0.1% | 5.0% |  |  |  |
| NMS | Non-English | 5.00 | 9.0% | 4.5% | 17.2% | 4.33 | 1.00 | 0.04 |
|  | English | 1.00 | 1.0% | 0.1% | 7.0% |  |  |  |

| SM 10. Inpatient deaths per 10,000 admissions according to English language | | | | | | | | |
| --- | --- | --- | --- | --- | --- | --- | --- | --- |
|  |  | n. studies | Estimate | Lower limit | Upper limit | Q-value | df (q) | P-value |
| Gastrointestinal | Non-English | 5 | 7.0 | 2.4 | 20.7 | 0.88 | 1 | 0.35 |
|  | English | 7 | 3.3 | 1.1 | 10.0 |  |  |  |
| Infectious | Non-English | 7 | 22.3 | 7.6 | 64.9 | 0.90 | 1 | 0.34 |
|  | English | 8 | 48.1 | 14.9 | 154.4 |  |  |  |
| Mental and beh | Non-English | 2 | 5.7 | 0.5 | 63.0 | 1.93 | 1 | 0.17 |
|  | English | 3 | 1.0 | 0.8 | 1.4 |  |  |  |
| Metabolic | Non-English | 5 | 5.1 | 2.2 | 11.9 | 0.35 | 1 | 0.56 |
|  | English | 8 | 3.4 | 1.2 | 9.5 |  |  |  |
| Neoplastic | Non-English | 6 | 24.0 | 16.5 | 35.0 | 1.07 | 1 | 0.30 |
|  | English | 8 | 8.0 | 1.0 | 62.0 |  |  |  |
| Neurological | Non-English | 8 | 4.0 | 2.3 | 7.1 | 0.16 | 1 | 0.69 |
|  | English | 8 | 3.3 | 1.5 | 7.4 |  |  |  |
| Respiratory | Non-English | 8 | 14.7 | 8.2 | 26.3 | 0.17 | 1 | 0.68 |
|  | English | 8 | 11.2 | 3.5 | 35.4 |  |  |  |
| Vascular | Non-English | 14 | 30.2 | 20.4 | 44.7 | 0.17 | 1 | 0.68 |
|  | English | 12 | 20.6 | 3.4 | 123.1 |  |  |  |
| Not Specified | Non-English | 4 | 26.6 | 3.9 | 178.7 | 4.00 | 1 | 0.05 |
|  | English | 6 | 3.0 | 1.2 | 7.7 |  |  |  |
| Total Natural | Non-English | 15 | 68.1 | 41.6 | 111.4 | 0.20 | 1 | 0.66 |
|  | English | 12 | 94.3 | 24.2 | 359.9 |  |  |  |
| Accident | Non-English | 9 | 8.0 | 3.8 | 16.8 | 0.59 | 1 | 0.44 |
|  | English | 5 | 4.8 | 1.7 | 13.8 |  |  |  |
| Homicide | Non-English | 1 | 8.0 | 1.1 | 56.4 | 0.67 | 1 | 0.41 |
|  | English | 1 | 2.5 | 0.4 | 17.8 |  |  |  |
| Suicide | Non-English | 12 | 7.4 | 3.9 | 14.1 | 1.58 | 1 | 0.01 |
|  | English | 8 | 3.8 | 1.7 | 8.6 |  |  |  |
| Total Unnatural | Non-English | 16 | 10.7 | 6.5 | 17.7 | 1.00 | 1 | 0.32 |
|  | English | 11 | 5.5 | 1.6 | 18.6 |  |  |  |
| Unknown | Non-English | 10 | 15.7 | 5.8 | 42.3 | 1.13 | 1 | 0.29 |
|  | English | 6 | 4.6 | 0.6 | 35.0 |  |  |  |
| Sudden | Non-English | 5 | 6.0 | 2.3 | 15.9 | 0.85 | 1 | 0.36 |
|  | English | 4 | 10.9 | 4.8 | 24.8 |  |  |  |
| Choking | Non-English | 3 | 1.3 | 0.2 | 10.3 | 0.55 | 1 | 0.46 |
|  | English | 3 | 3.6 | 0.7 | 17.1 |  |  |  |
| ECT | Non-English | 3 | 2.4 | 0.8 | 7.1 | 0.70 | 1 | 0.40 |
|  | English | 1 | 0.9 | 0.1 | 6.7 |  |  |  |
| NMS | Non-English | 5 | 3.6 | 2.0 | 6.5 | 1.79 | 1 | 0.18 |
|  | English | 1 | 0.9 | 0.1 | 6.2 |  |  |  |
| Total death | Non-English | 15 | 119.7 | 83.0 | 172.4 | 0.04 | 1 | 0.84 |
|  | English | 11 | 102.6 | 23.7 | 432.1 |  |  |  |

| SM 11. Inpatient deaths per 100,000 person years according to English language | | | | | | | | |
| --- | --- | --- | --- | --- | --- | --- | --- | --- |
|  |  | n. studies | Estimate | Lower limit | Upper limit | Q-value | df (q) | P-value |
| Gastrointestinal | Non-English | 5 | 109.5 | 0.0 | 242.4 | 0.02 | 1 | 0.89 |
|  | English | 6 | 119.6 | 52.5 | 186.7 |  |  |  |
| Infectious | Non-English | 6 | 532.2 | 304.3 | 760.2 | 3.41 | 1 | 0.07 |
|  | English | 9 | 853.5 | 599.7 | 1107.3 |  |  |  |
| Mental and beh | Non-English | 1 | 297.6 | 215.1 | 380.1 | 6.07 | 1 | 0.01 |
|  | English | 3 | 118.1 | 1.5 | 234.7 |  |  |  |
| Metabolic | Non-English | 4 | 50.0 | 0.0 | 124.4 | 1.59 | 1 | 0.21 |
|  | English | 7 | 125.8 | 34.5 | 217.1 |  |  |  |
| Neoplastic | Non-English | 6 | 214.9 | 59.2 | 370.7 | 1.24 | 1 | 0.73 |
|  | English | 10 | 184.0 | 110.2 | 257.8 |  |  |  |
| Neurological | Non-English | 6 | 162.5 | 27.1 | 297.8 | 0.00 | 1 | 0.98 |
|  | English | 7 | 160.0 | 76.1 | 243.8 |  |  |  |
| Respiratory | Non-English | 7 | 618.3 | 338.3 | 898.3 | 3.60 | 1 | 0.06 |
|  | English | 8 | 320.2 | 192.3 | 448.0 |  |  |  |
| Vascular | Non-English | 10 | 1327.2 | 748.1 | 1906.4 | 3.56 | 1 | 0.06 |
|  | English | 13 | 733.0 | 518.9 | 947.2 |  |  |  |
| Not Specified | Non-English | 5 | 964.9 | 735.6 | 1194.2 | 30.09 | 1 | <.001 |
|  | English | 7 | 226.2 | 95.6 | 356.9 |  |  |  |
| Total Natural | Non-English | 13 | 2574.8 | 1496.9 | 3652.7 | 1.15 | 1 | 0.29 |
|  | English | 12 | 3282.9 | 2561.0 | 4004.9 |  |  |  |
| Accident | Non-English | 7 | 94.6 | 59.1 | 130.1 | 0.02 | 1 | 0.88 |
|  | English | 5 | 86.4 | 0.0 | 182.5 |  |  |  |
| Homicide | Non-English | 2 | 2.3 | -0.7 | 5.3 | 0.80 | 1 | 0.37 |
|  | English | 1 | 21.7 | -20.9 | 64.3 |  |  |  |
| Suicide | Non-English | 9 | 181.7 | 112.4 | 251.0 | 0.81 | 1 | 0.37 |
|  | English | 8 | 137.1 | 68.0 | 206.1 |  |  |  |
| Total Unnatural | Non-English | 13 | 234.9 | 144.4 | 325.5 | 1.02 | 1 | 0.31 |
|  | English | 11 | 174.9 | 101.4 | 248.4 |  |  |  |
| Unknown | Non-English | 8 | 958.1 | 437.2 | 1479.0 | 7.91 | 1.00 | 0.01 |
|  | English | 5 | 178.9 | 25.8 | 332.1 |  |  |  |
| Sudden | Non-English | 3 | 385.7 | 73.7 | 697.7 | 0.01 | 1.00 | 0.95 |
|  | English | 5 | 399.2 | 159.0 | 639.4 |  |  |  |
| Choking | Non-English | 2 | 142.7 | 27.7 | 257.7 | 1.57 | 1.00 | 0.21 |
|  | English | 3 | 62.0 | 9.7 | 114.3 |  |  |  |
| ECT | Non-English | 1 | 41.4 | 0.0 | 122.6 | 0.38 | 1.00 | 0.54 |
|  | English | 1 | 14.3 | 0.0 | 42.5 |  |  |  |
| NMS | Non-English | 3 | 108.0 | 0.0 | 293.7 | 0.86 | 1.00 | 0.36 |
|  | English | 1 | 18.7 | 0.0 | 55.3 |  |  |  |
| Total death | Non-English | 13 | 3818.5 | 2175.2 | 5461.8 | 0.01 | 1.00 | 0.94 |
|  | English | 11 | 3892.1 | 2999.5 | 4784.7 |  |  |  |

| SM 12. Adjusted proportion of mortalities in psychiatric hospitals and heterogeneity not explained by publication date | | | | | |  |  |  |  |  |  |  |
| --- | --- | --- | --- | --- | --- | --- | --- | --- | --- | --- | --- | --- |
|  | Tweedie and Duval’s Adjusted Estimate | | | | Heterogeneity unexplained by meta-regression on date |  |  |  |  |  |  |  |
|  | Trimmed studies n. | Estimate (%) | Lower limit  (%) | Upper limit  (%) | I-square |  |  |  |  |  |  |  |
| Gastrointestinal | - | - | - | - | 73.5 |  |  |  |  |  |  |  |
| Infectious | - | - | - | - | 97.8 |  |  |  |  |  |  |  |
| Ment. & Beh. | - | - | - | - | 77.4 |  |  |  |  |  |  |  |
| Metabolic | - | - | - | - | 83.4 |  |  |  |  |  |  |  |
| Neoplastic | - | - | - | - | 87.3 |  |  |  |  |  |  |  |
| Neurological | 5 | 8.6 | 6.4 | 11.6 | 47.4 |  |  |  |  |  |  |  |
| Respiratory | - | - | - | - | 98.1 |  |  |  |  |  |  |  |
| Vascular | - | - | - | - | 96.7 |  |  |  |  |  |  |  |
| Not Specified | 2 | 12.7 | 8.3 | 18.9 | 96.7 |  |  |  |  |  |  |  |
| **Total Natural** | **9** | **73.3** | **66.3** | **79.3** | **98.4** |  |  |  |  |  |  |  |
| Accident | - | - | - | - | 90.2 |  |  |  |  |  |  |  |
| Homicide | - | - | - | - | - |  |  |  |  |  |  |  |
| Suicide |  | - | - | - | 94.4 |  |  |  |  |  |  |  |
| **Total Unnatural** | **-** | **-** | **-** | **-** | **98.4** |  |  |  |  |  |  |  |
|  |  |  |  |  |  |  |  |  |  |  |  |  |
|  |  |  |  |  |  |  |  |  |  |  |  |  |
|  |  |  |  |  |  |  |  |  |  |  |  |  |
|  |  |  |  |  |  |  |  |  |  |  |  |  |
|  |  |  |  |  |  |  |  |  |  |  |  |  |

|  |  |  |  |  |  |  |  |  |  |  |  |  |
| --- | --- | --- | --- | --- | --- | --- | --- | --- | --- | --- | --- | --- |

| SM 13. Adjusted mortalities in psychiatric hospitals per 10,000 admissions and heterogeneity not explained by publication date | | | | | |
| --- | --- | --- | --- | --- | --- |
|  | Tweedie and Duval’s Adjusted Estimate | | | | Heterogeneity unexplained by meta-regression on date |
|  | Trimmed studies n. | Estimate | Lower limit | Upper limit | I-square |
| Gastrointestinal | - | - | - | - | 87.5 |
| Infectious | - | - | - | - | 98.8 |
| Ment. & Beh. | - | - | - | - | 34.0 |
| Metabolic | - | - | - | - | 87.2 |
| Neoplastic | - | - | - | - | 98.4 |
| Neurological | - | - | - | - | 79.2 |
| Respiratory | 0 | - | - | - | 96.0 |
| Vascular | 6 | 44.2 | 26.4 | 73.9 | 99.5 |
| Not Specified | - | - | - | - | 96.8 |
| **Total Natural** | **0** | **-** | **-** | **-** | **99.5** |
| Accident | 0 | - | - | - | 97.6 |
| Homicide | 0 | - | - | - | - |
| Suicide | - | - | - | - | 97.0 |
| **Total Unnatural** | **5** | **12.3** | **7.4** | **20.4** | **97.6** |
| **Total Deaths** | **0** | **-** | **-** | **-** | **99.7** |

|  |  |  |  | |  |  |  | |  |  |  |  |  |  |  |
| --- | --- | --- | --- | --- | --- | --- | --- | --- | --- | --- | --- | --- | --- | --- | --- |
| SM 14. Adjusted mortalities in psychiatric hospitals per 100,000 person years heterogeneity not explained by publication date | | | | | | | |  |  |  |  |  |  |  |  |
|  | Tweedie and Duval’s Adjusted Estimate | | | | | Heterogeneity unexplained by meta-regression on date | |  |  |  |  |  |  |  |  |
|  | Trimmed studies n. | Estimate | | Lower limit | Upper limit | I-square | |  |  |  |  |  |  |  |  |
| Gastrointestinal | 4 | 97 | | 40 | 156 | 85.8 | |  |  |  |  |  |  |  |  |
| Infectious | 2 | 615 | | 473 | 757 | 98.0 | |  |  |  |  |  |  |  |  |
| Ment. & Beh. | - | - | | - | - | 96.3 | |  |  |  |  |  |  |  |  |
| Metabolic | 6 | 21 | | 0 | 45 | 85.2 | |  |  |  |  |  |  |  |  |
| Neoplastic | 4 | 168 | | 108 | 227 | 95.0 | |  |  |  |  |  |  |  |  |
| Neurological | 5 | 98 | | 43 | 182 | 77.8 | |  |  |  |  |  |  |  |  |
| Respiratory | 3 | 282 | | 194 | 371 | 98.0 | |  |  |  |  |  |  |  |  |
| Vascular | 1 | 877 | | 656 | 1097 | 98.6 | |  |  |  |  |  |  |  |  |
| Not Specified | 3 | 370 | | 176 | 564 | 97.6 | |  |  |  |  |  |  |  |  |
| **Total Natural** | **3** | **4333** | | **2620** | **6046** | **99.5** | |  |  |  |  |  |  |  |  |
| Accident | 4 | 83 | | 35 | 131 | 77.0 | |  |  |  |  |  |  |  |  |
| Homicide | 2 | 2 | | 0.0 | 5 | - | |  |  |  |  |  |  |  |  |
| Suicide | 7 | 55 | | 14 | 96 | 93.2 | |  |  |  |  |  |  |  |  |
| **Total Unnatural** | **8** | **111** | | **64** | **158** | **94.1** | |  |  |  |  |  |  |  |  |
| **Total Deaths** | **1** | **5398** | | **3163** | **7632** | **99.6** | |  |  |  |  |  |  |  |  |
